# Supplementary material for: Direct-Acting Oral Anticoagulants: A Resident-Based Workshop to Improve Knowledge and Confidence
Source: MedEdPORTAL. 2020 Sep 30;16:10981. doi: 10.15766/mep_2374-8265.10981 (PMC7526504; doi:10.15766/mep_2374-8265.10981)
Supplement: Supplementary file 1 — Preworkshop MCQ Students.docxDOAC PowerPoint.pptDOAC Indications and Dosing Case.docxDOAC Monitoring and Reversal Case.docxDOAC Dosing Elderly Case.docxDOAC Peri-procedural Case.docxPostworkshop MCQ and Confidence Survey Students.docxPostworkshop MCQ Facilitators.docx [file mep_2374-8265.10981-s001.zip › A. Preworkshop MCQ Students.docx]

**DOAC Questions**

1. 76 yo female with HTN and DM presents to clinic with new afib. BP 130/80, HR 80, wt 70 kg. CrCl 45. What treatment would you recommend?
2. No treatment
3. Edoxaban 30 mg daily
4. Warfarin 5 mg daily
5. Rivaroxaban 5 mg daily
6. Apixaban 2.5 mg bid
7. 60 yo male p/w acute iliofemoral DVT. Patient prefers an anticoagulant with an antidote. Which of the following does NOT have an FDA-approved antidote?
8. Edoxaban
9. Warfarin
10. Dabigatran
11. Heparin
12. Apixaban
13. 82 yo woman p/w new afib. CrCl 14. Which anticoagulant would you start?
14. Heparin gtt + warfarin
15. Enoxaparin q12h + warfarin
16. Apixaban 2.5 mg bid
17. Rivaroxaban 15 mg daily
18. 52 yo male on rivaroxaban 15 mg QHS and CrCl 45 planning lumbar epidural steroid injection. How do you approach peri-procedural anticoagulation?
19. Continue rivaroxaban
20. Skip 1 dose rivaroxaban
21. Skip 2 doses rivaroxaban
22. Skip 3 doses rivaroxaban and start enoxaparin 48 hour before procedure
