# Supplementary material for: Direct-Acting Oral Anticoagulants: A Resident-Based Workshop to Improve Knowledge and Confidence
Source: MedEdPORTAL. 2020 Sep 30;16:10981. doi: 10.15766/mep_2374-8265.10981 (PMC7526504; doi:10.15766/mep_2374-8265.10981)
Supplement: Supplementary file 1 — Preworkshop MCQ Students.docxDOAC PowerPoint.pptDOAC Indications and Dosing Case.docxDOAC Monitoring and Reversal Case.docxDOAC Dosing Elderly Case.docxDOAC Peri-procedural Case.docxPostworkshop MCQ and Confidence Survey Students.docxPostworkshop MCQ Facilitators.docx [file mep_2374-8265.10981-s001.zip › B. DOAC PowerPoint.ppt]

## Slide 1
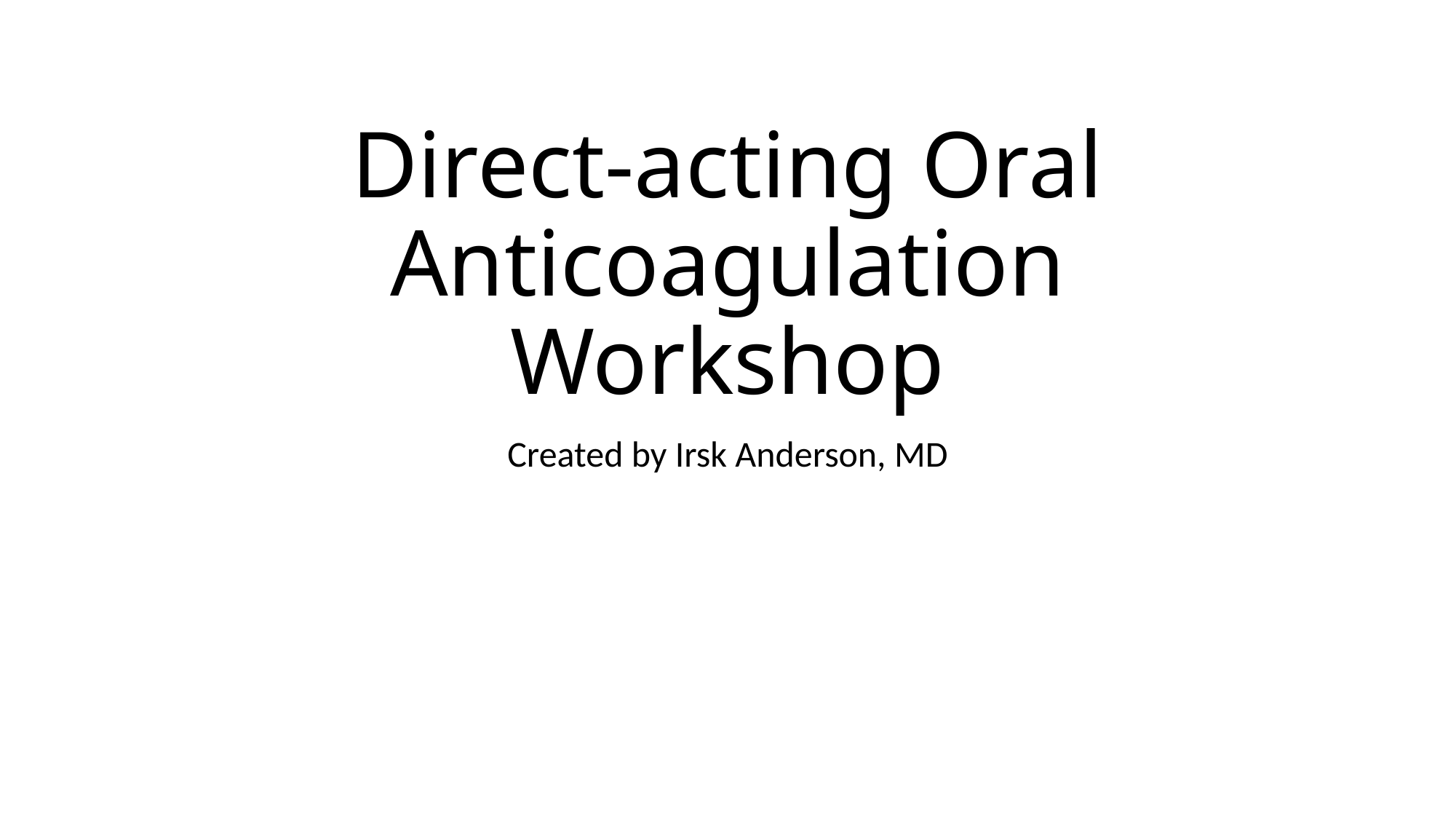

# Direct-acting Oral Anticoagulation Workshop
Created by Irsk Anderson, MD

## Slide 2
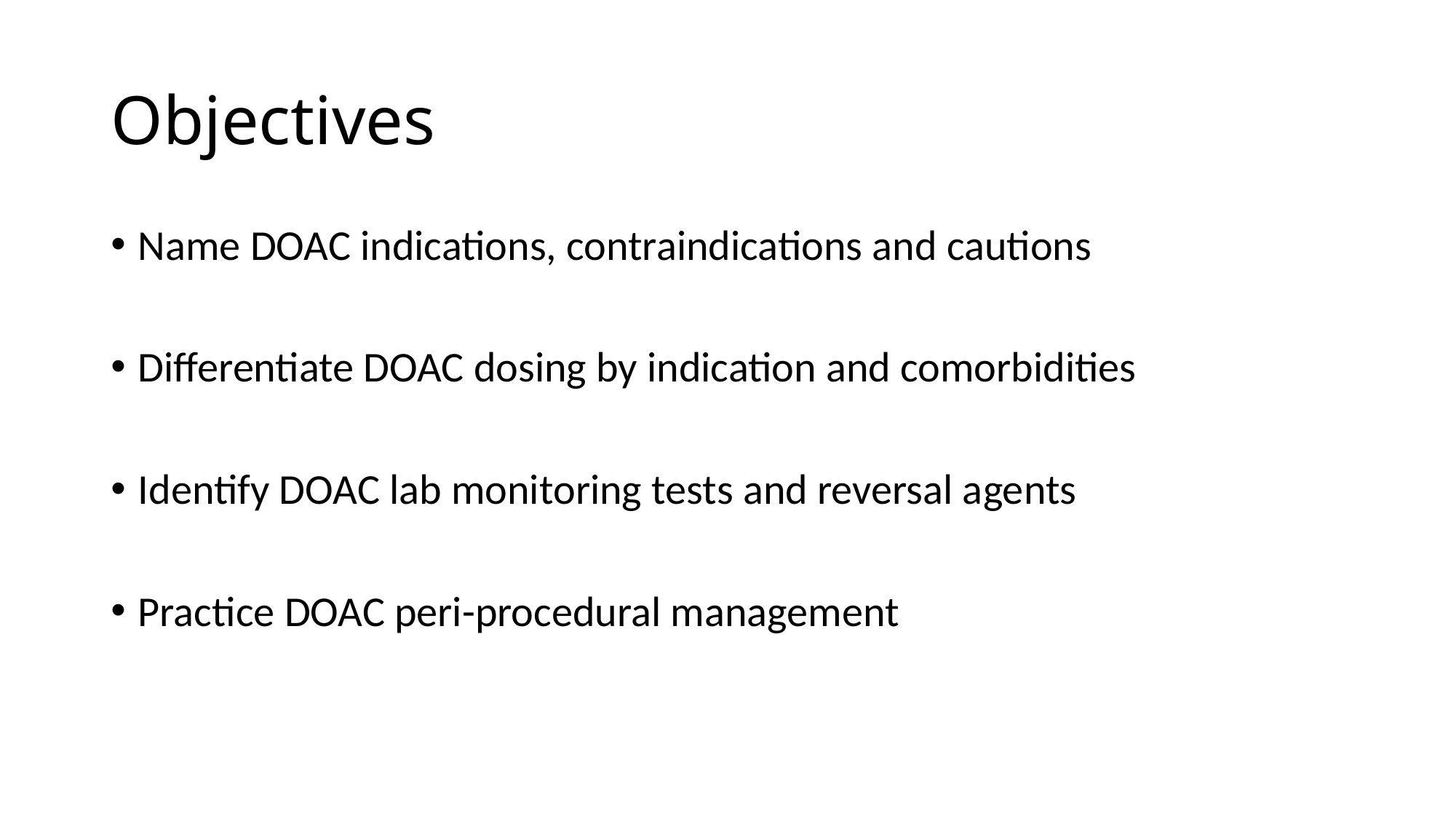

# Objectives
Name DOAC indications, contraindications and cautions
Differentiate DOAC dosing by indication and comorbidities
Identify DOAC lab monitoring tests and reversal agents
Practice DOAC peri-procedural management

## Slide 3
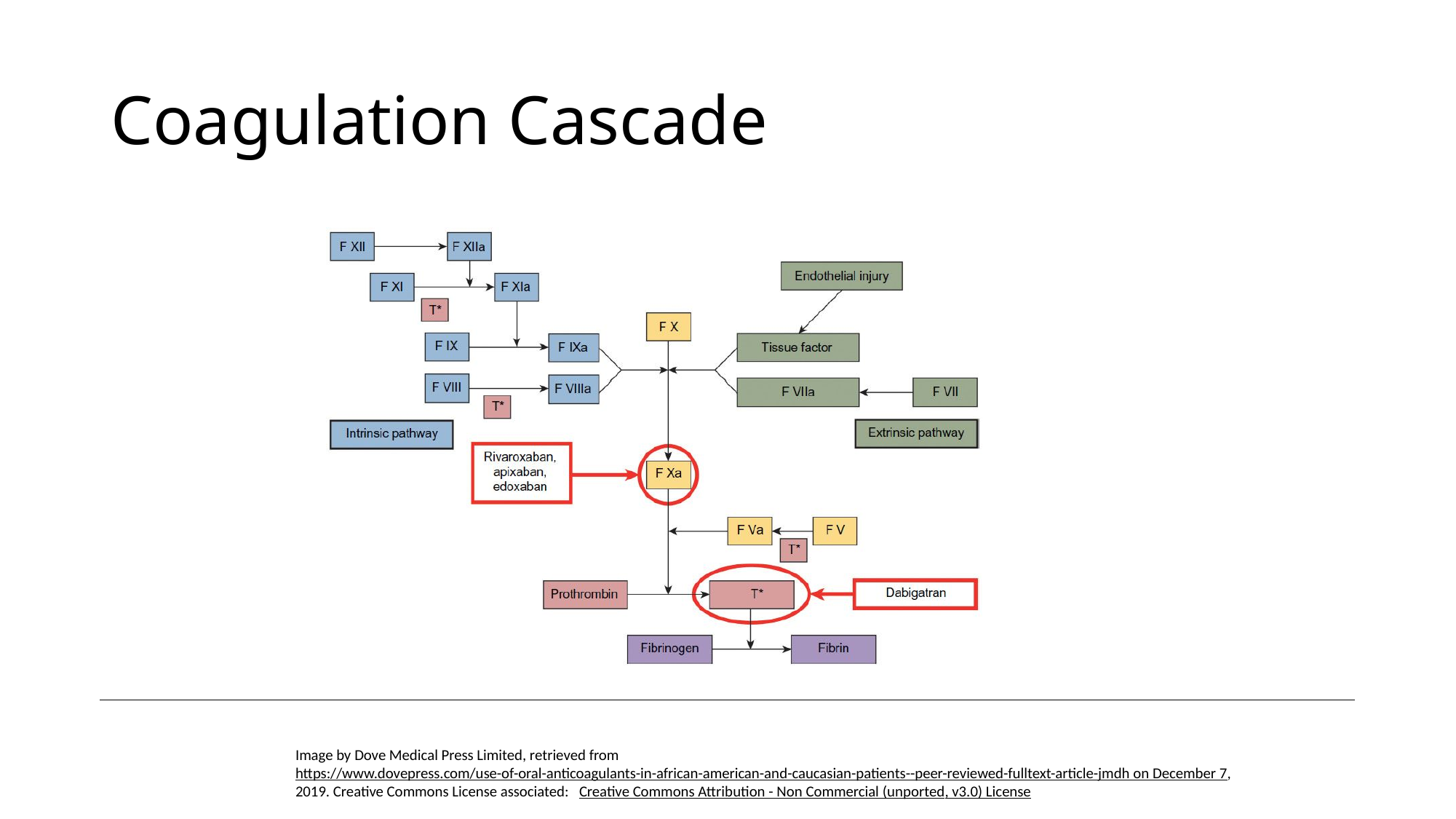

# Coagulation Cascade
| | |
| --- | --- |
Image by Dove Medical Press Limited, retrieved from https://www.dovepress.com/use-of-oral-anticoagulants-in-african-american-and-caucasian-patients--peer-reviewed-fulltext-article-jmdh on December 7, 2019. Creative Commons License associated: Creative Commons Attribution - Non Commercial (unported, v3.0) License

## Slide 4
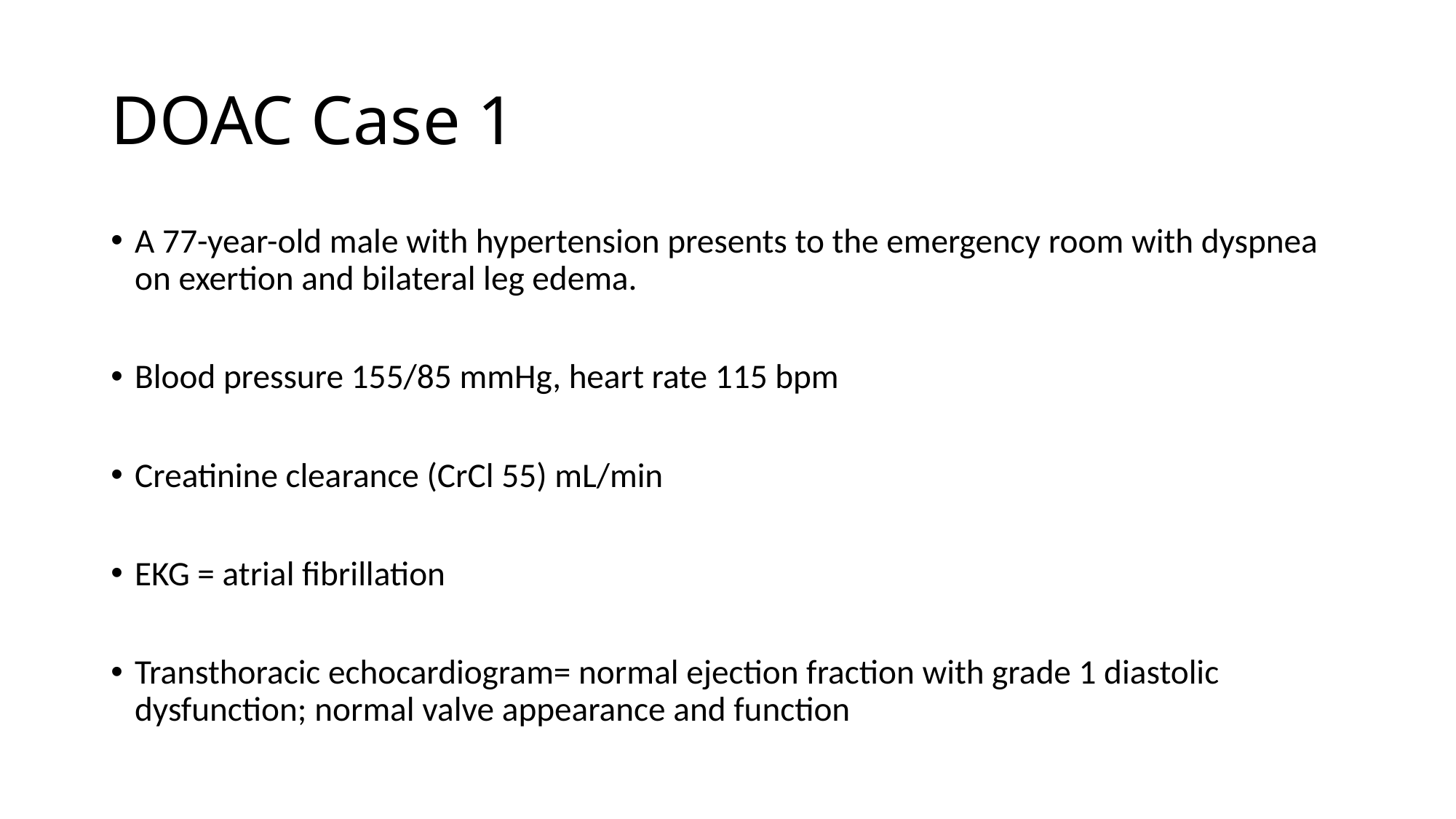

# DOAC Case 1
A 77-year-old male with hypertension presents to the emergency room with dyspnea on exertion and bilateral leg edema.
Blood pressure 155/85 mmHg, heart rate 115 bpm
Creatinine clearance (CrCl 55) mL/min
EKG = atrial fibrillation
Transthoracic echocardiogram= normal ejection fraction with grade 1 diastolic dysfunction; normal valve appearance and function

## Slide 5
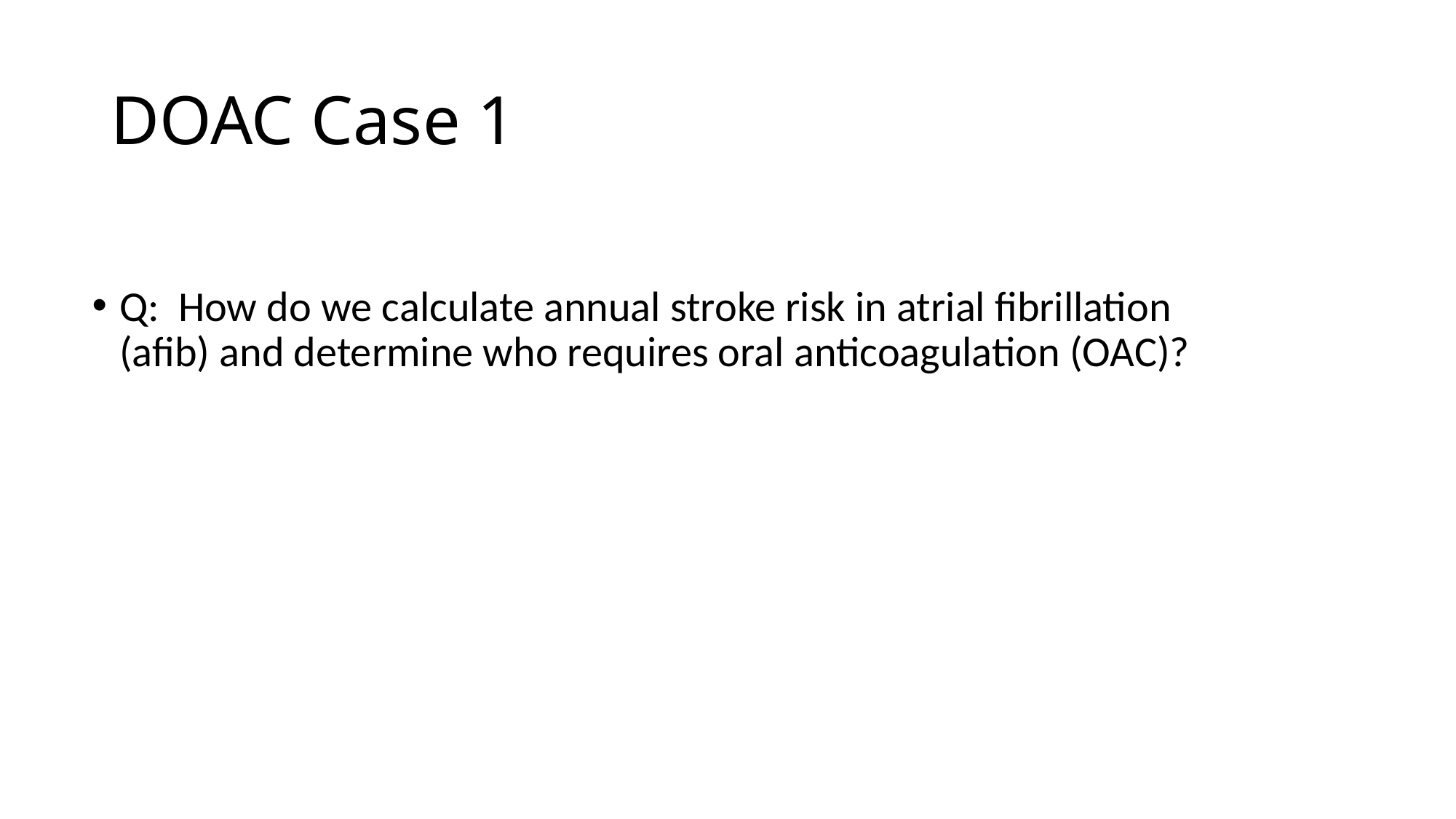

# DOAC Case 1
Q: How do we calculate annual stroke risk in atrial fibrillation (afib) and determine who requires oral anticoagulation (OAC)?

## Slide 6
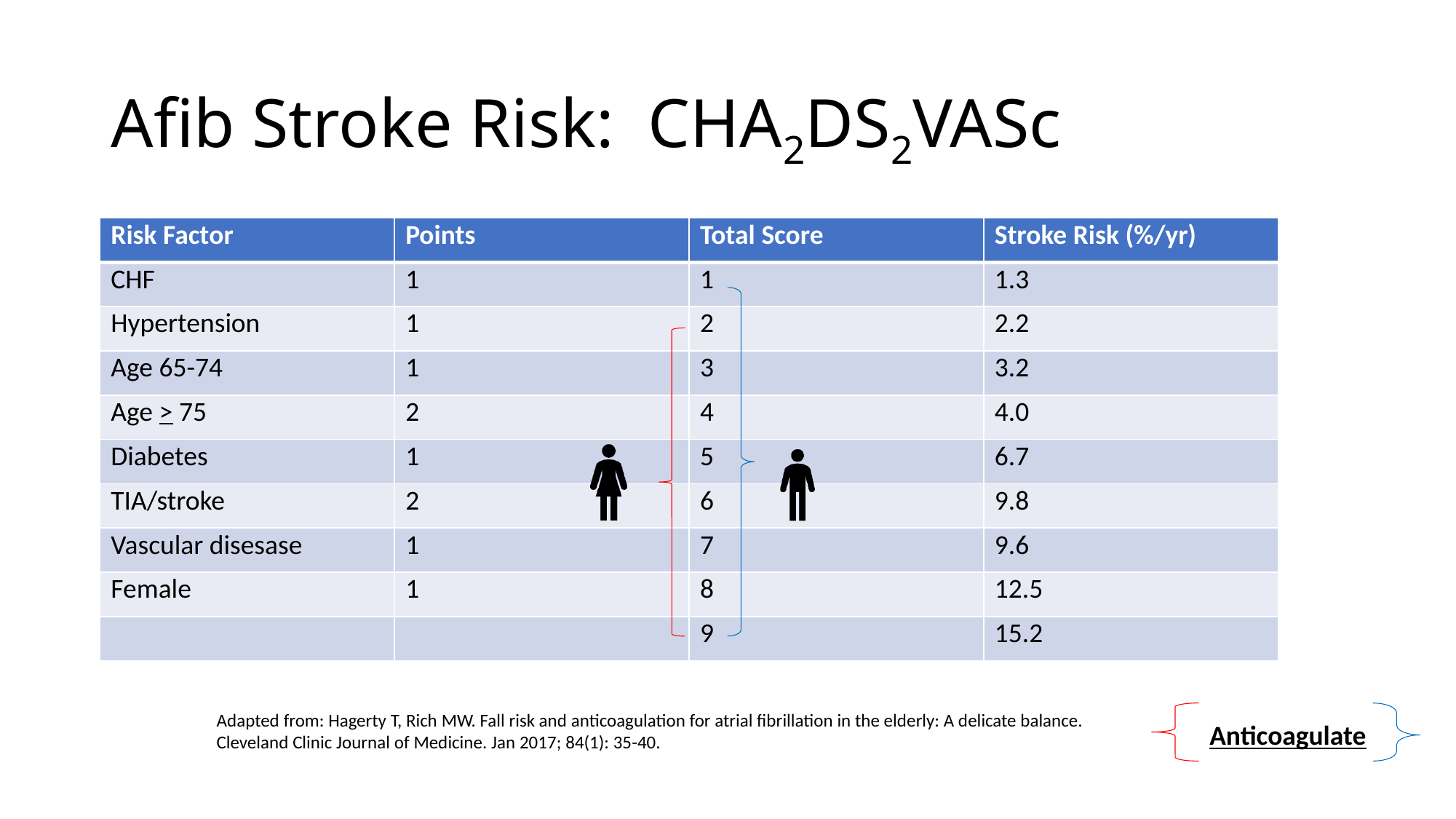

# Afib Stroke Risk: CHA2DS2VASc
| Risk Factor | Points | Total Score | Stroke Risk (%/yr) |
| --- | --- | --- | --- |
| CHF | 1 | 1 | 1.3 |
| Hypertension | 1 | 2 | 2.2 |
| Age 65-74 | 1 | 3 | 3.2 |
| Age > 75 | 2 | 4 | 4.0 |
| Diabetes | 1 | 5 | 6.7 |
| TIA/stroke | 2 | 6 | 9.8 |
| Vascular disesase | 1 | 7 | 9.6 |
| Female | 1 | 8 | 12.5 |
| | | 9 | 15.2 |
Adapted from: Hagerty T, Rich MW. Fall risk and anticoagulation for atrial fibrillation in the elderly: A delicate balance. Cleveland Clinic Journal of Medicine. Jan 2017; 84(1): 35-40.
Anticoagulate

## Slide 7
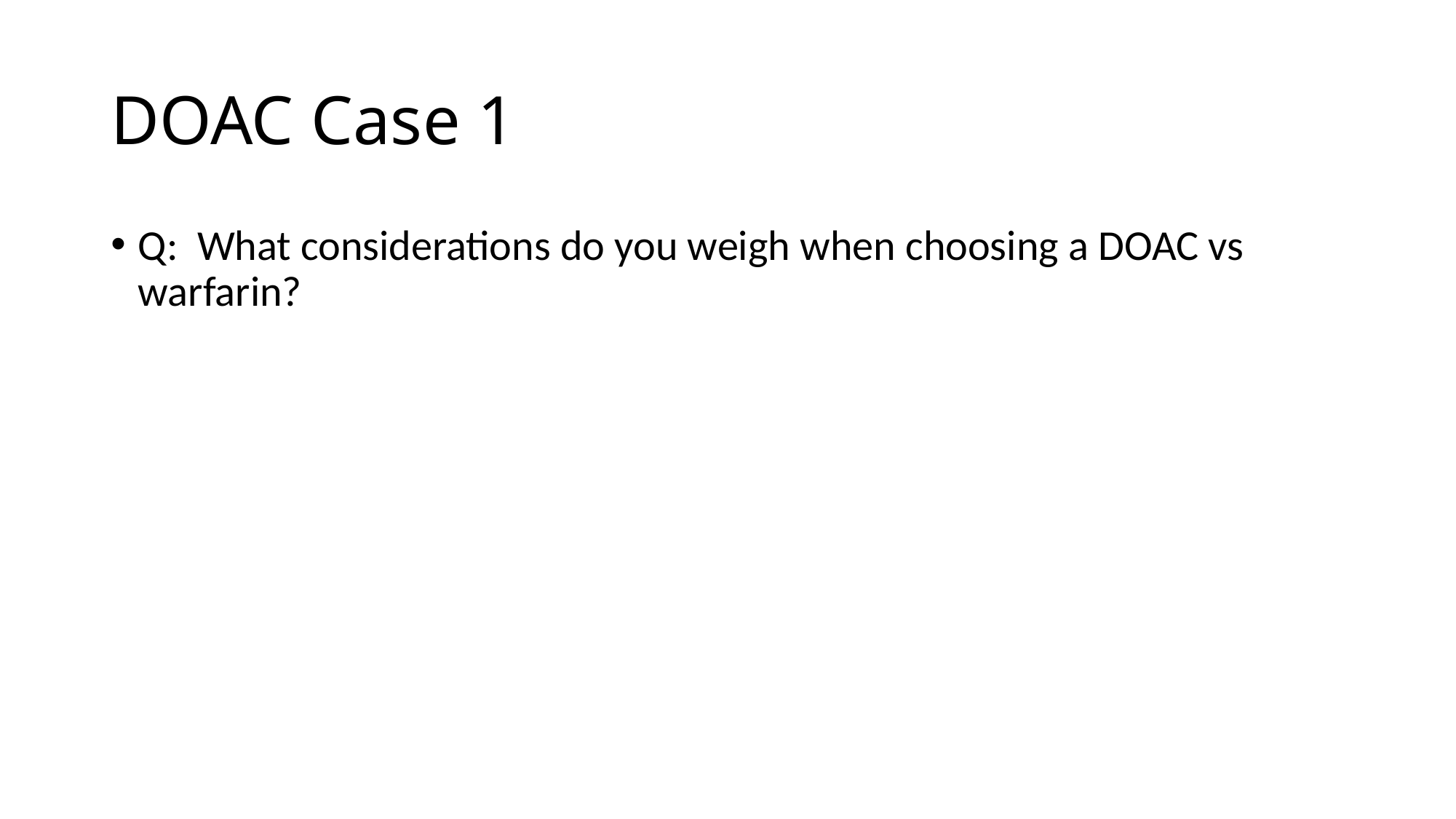

# DOAC Case 1
Q: What considerations do you weigh when choosing a DOAC vs warfarin?

## Slide 8
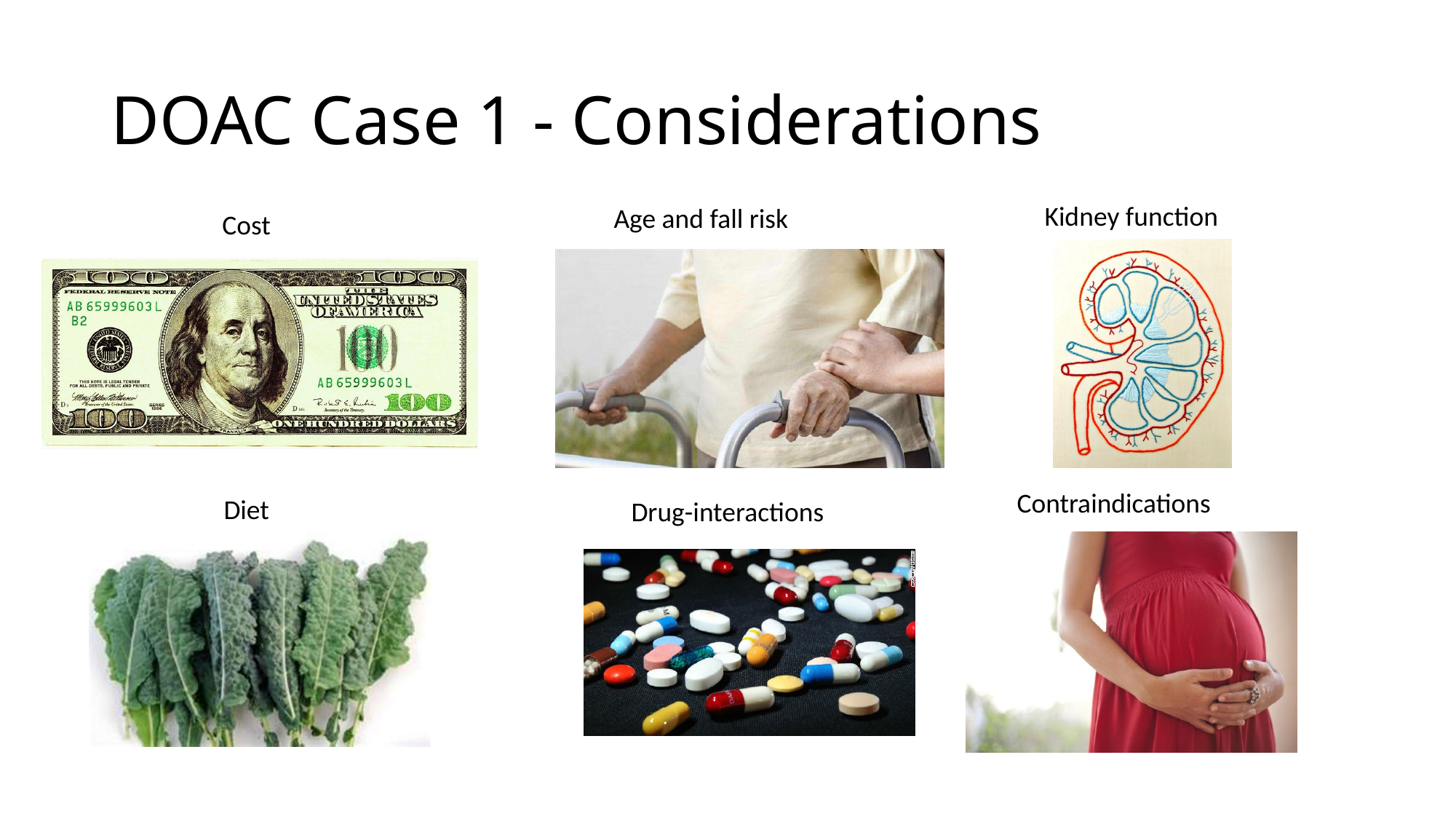

# DOAC Case 1 - Considerations
Kidney function
Age and fall risk
Cost
Contraindications
Diet
Drug-interactions

## Slide 9
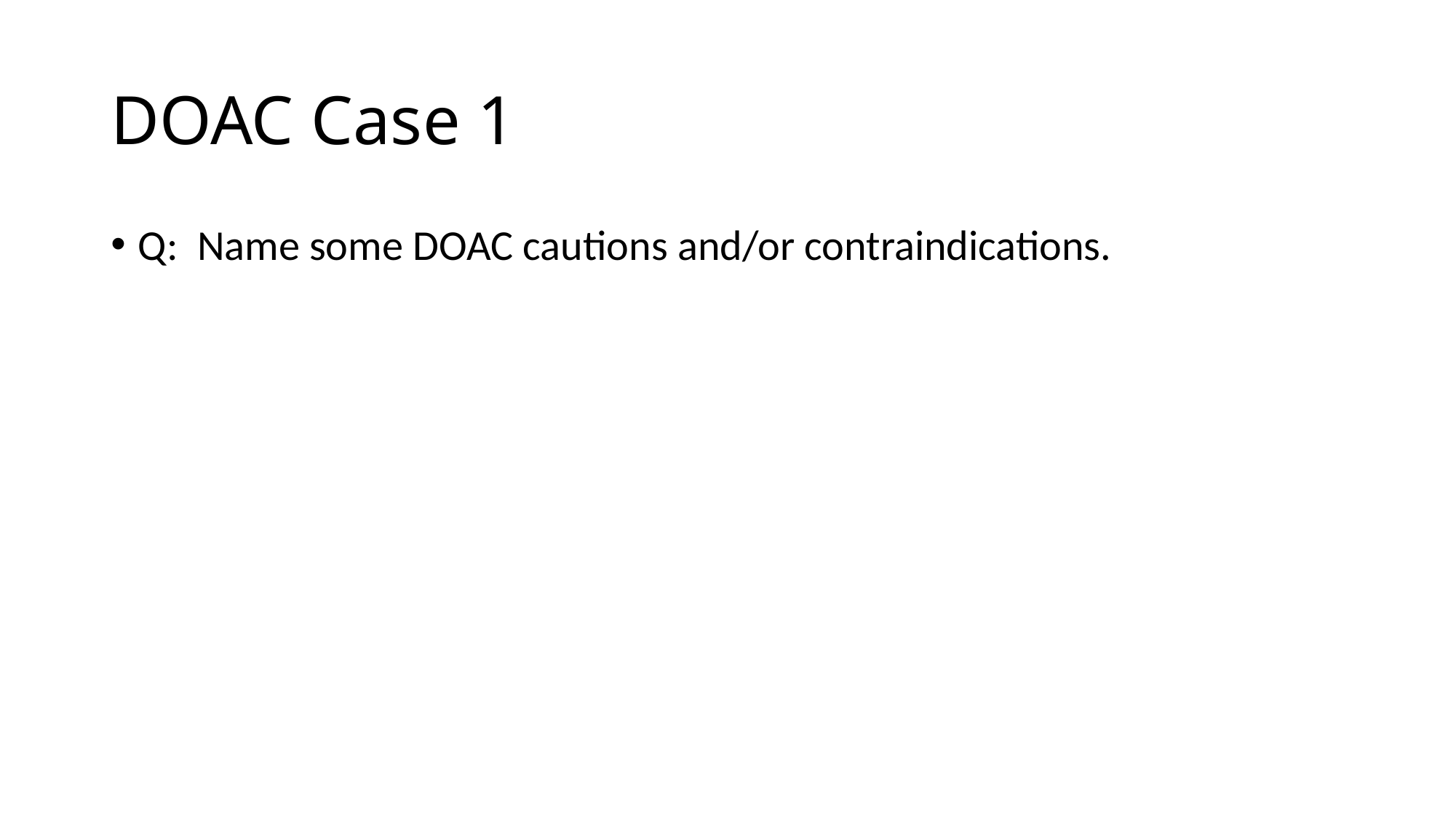

# DOAC Case 1
Q: Name some DOAC cautions and/or contraindications.

## Slide 10
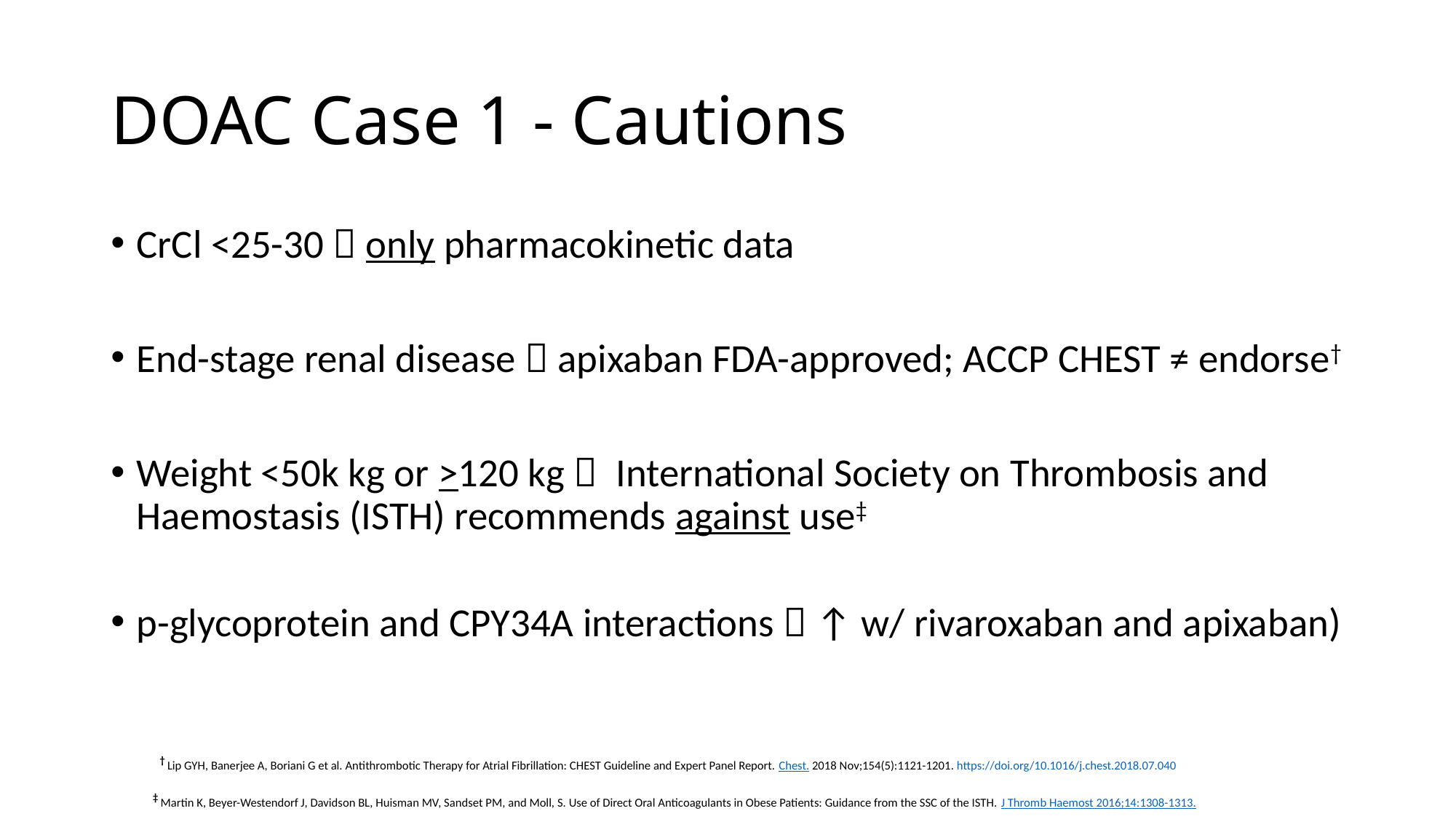

# DOAC Case 1 - Cautions
CrCl <25-30  only pharmacokinetic data
End-stage renal disease  apixaban FDA-approved; ACCP CHEST ≠ endorse†
Weight <50k kg or >120 kg  International Society on Thrombosis and Haemostasis (ISTH) recommends against use‡
p-glycoprotein and CPY34A interactions  ↑ w/ rivaroxaban and apixaban)
† Lip GYH, Banerjee A, Boriani G et al. Antithrombotic Therapy for Atrial Fibrillation: CHEST Guideline and Expert Panel Report. Chest. 2018 Nov;154(5):1121-1201. https://doi.org/10.1016/j.chest.2018.07.040
‡ Martin K, Beyer-Westendorf J, Davidson BL, Huisman MV, Sandset PM, and Moll, S. Use of Direct Oral Anticoagulants in Obese Patients: Guidance from the SSC of the ISTH. J Thromb Haemost 2016;14:1308-1313.

## Slide 11
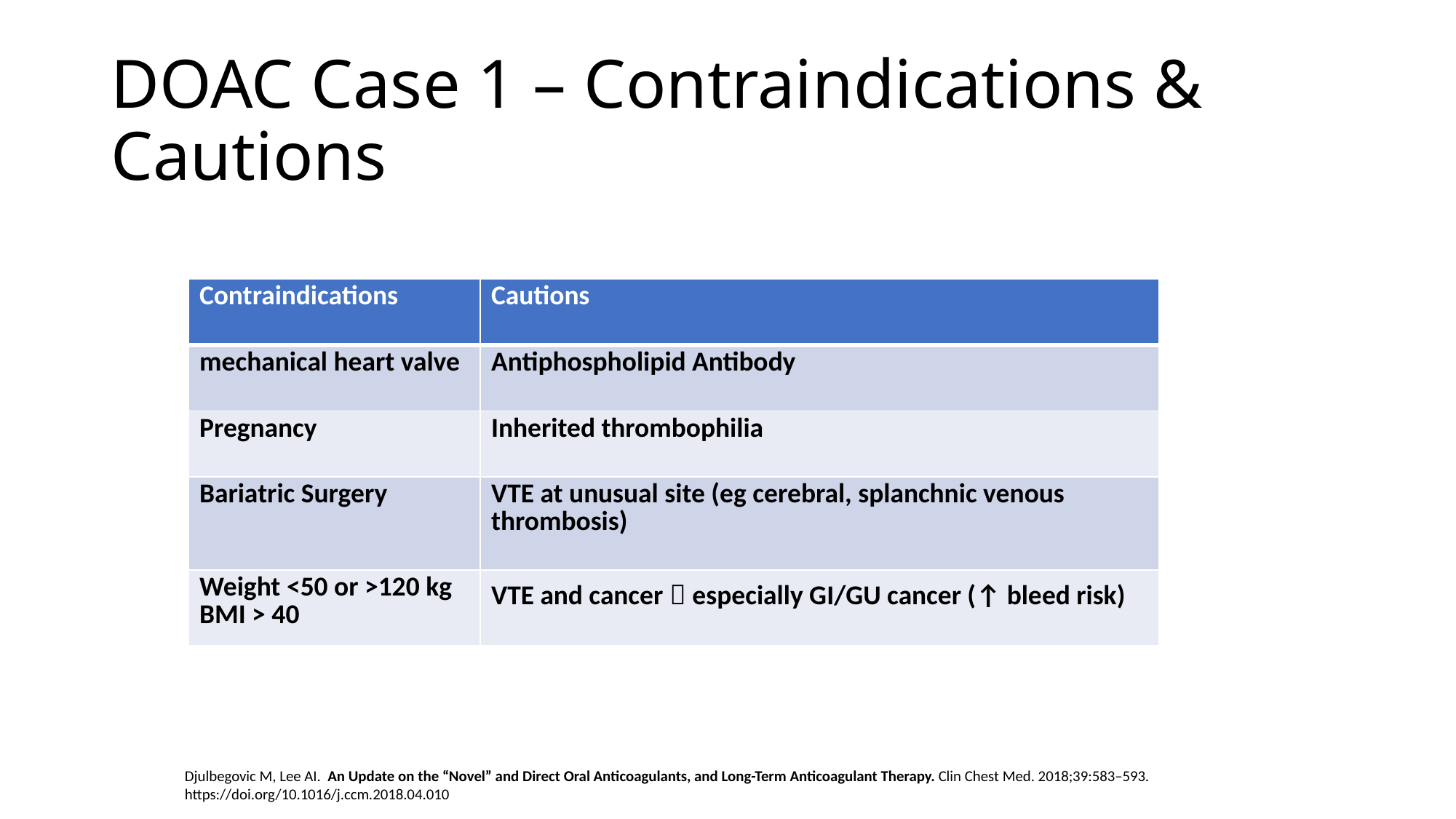

# DOAC Case 1 – Contraindications & Cautions
| Contraindications | Cautions |
| --- | --- |
| mechanical heart valve | Antiphospholipid Antibody |
| Pregnancy | Inherited thrombophilia |
| Bariatric Surgery | VTE at unusual site (eg cerebral, splanchnic venous thrombosis) |
| Weight <50 or >120 kg BMI > 40 | VTE and cancer  especially GI/GU cancer (↑ bleed risk) |
Djulbegovic M, Lee AI. An Update on the “Novel” and Direct Oral Anticoagulants, and Long-Term Anticoagulant Therapy. Clin Chest Med. 2018;39:583–593. https://doi.org/10.1016/j.ccm.2018.04.010

## Slide 12
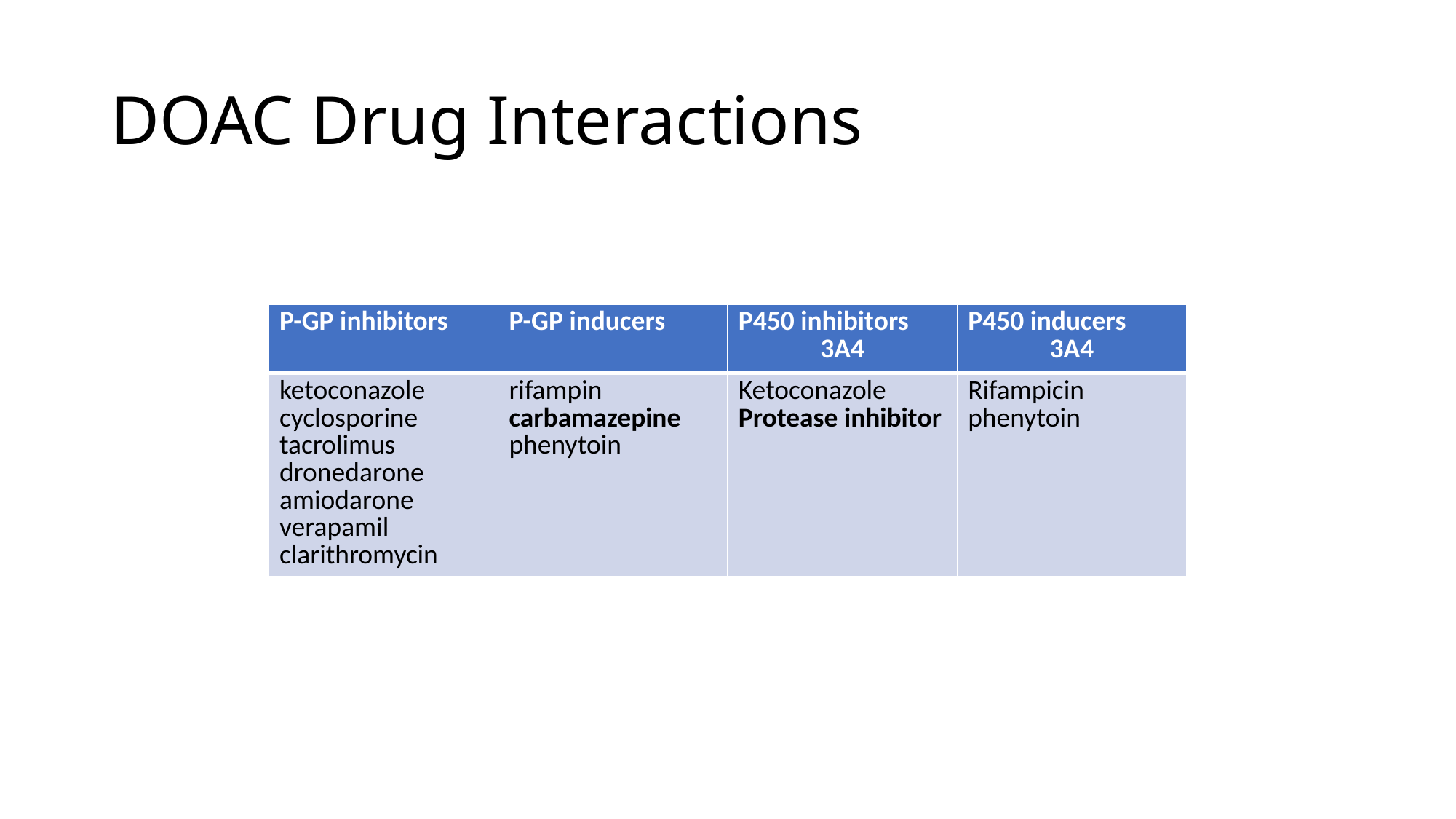

# DOAC Drug Interactions
| P-GP inhibitors | P-GP inducers | P450 inhibitors 3A4 | P450 inducers 3A4 |
| --- | --- | --- | --- |
| ketoconazole cyclosporine tacrolimus dronedarone amiodarone verapamil clarithromycin | rifampin carbamazepine phenytoin | Ketoconazole Protease inhibitor | Rifampicin phenytoin |

## Slide 13
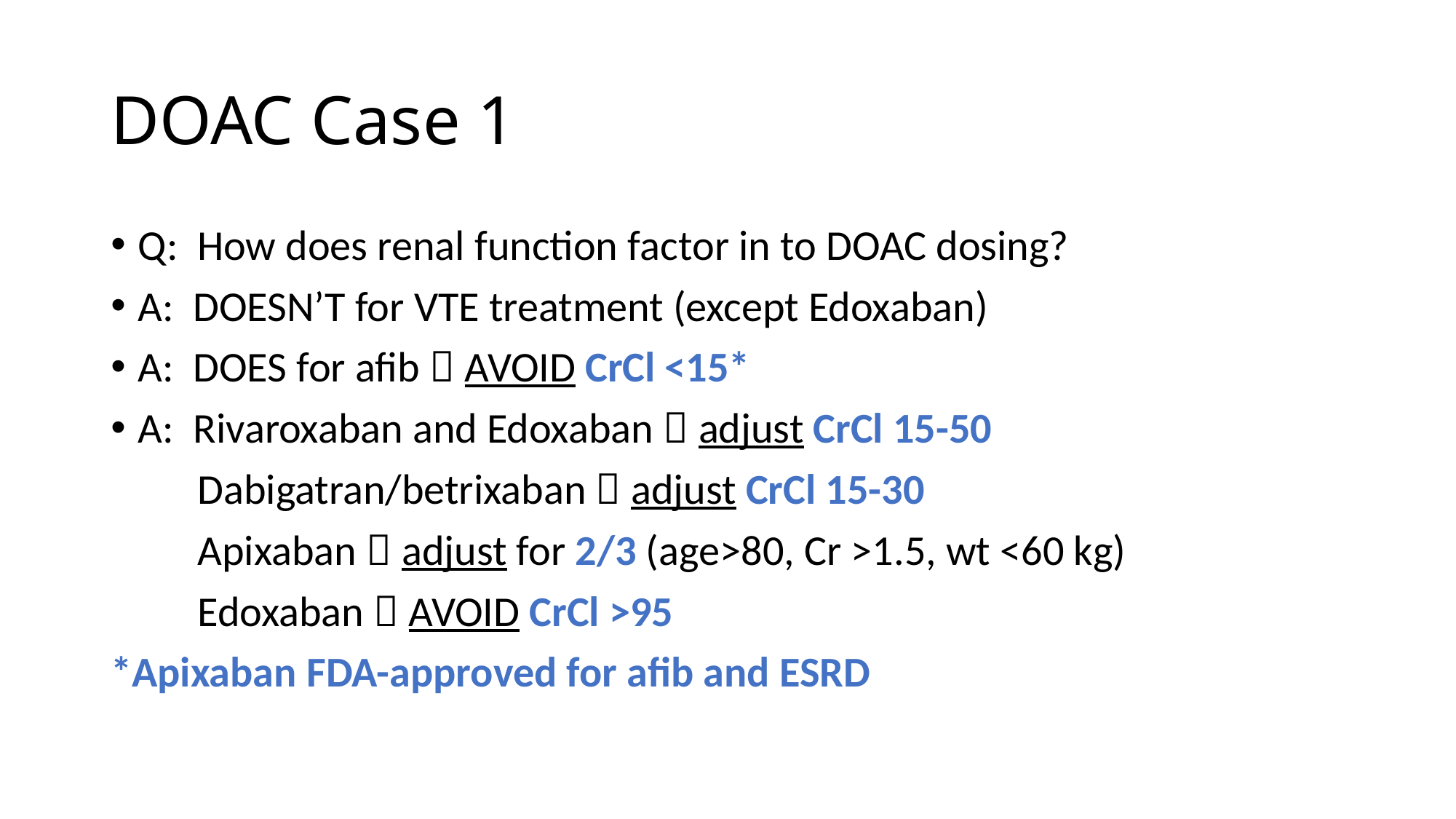

# DOAC Case 1
Q: How does renal function factor in to DOAC dosing?
A: DOESN’T for VTE treatment (except Edoxaban)
A: DOES for afib  AVOID CrCl <15*
A: Rivaroxaban and Edoxaban  adjust CrCl 15-50
 Dabigatran/betrixaban  adjust CrCl 15-30
 Apixaban  adjust for 2/3 (age>80, Cr >1.5, wt <60 kg)
 Edoxaban  AVOID CrCl >95
*Apixaban FDA-approved for afib and ESRD

## Slide 14
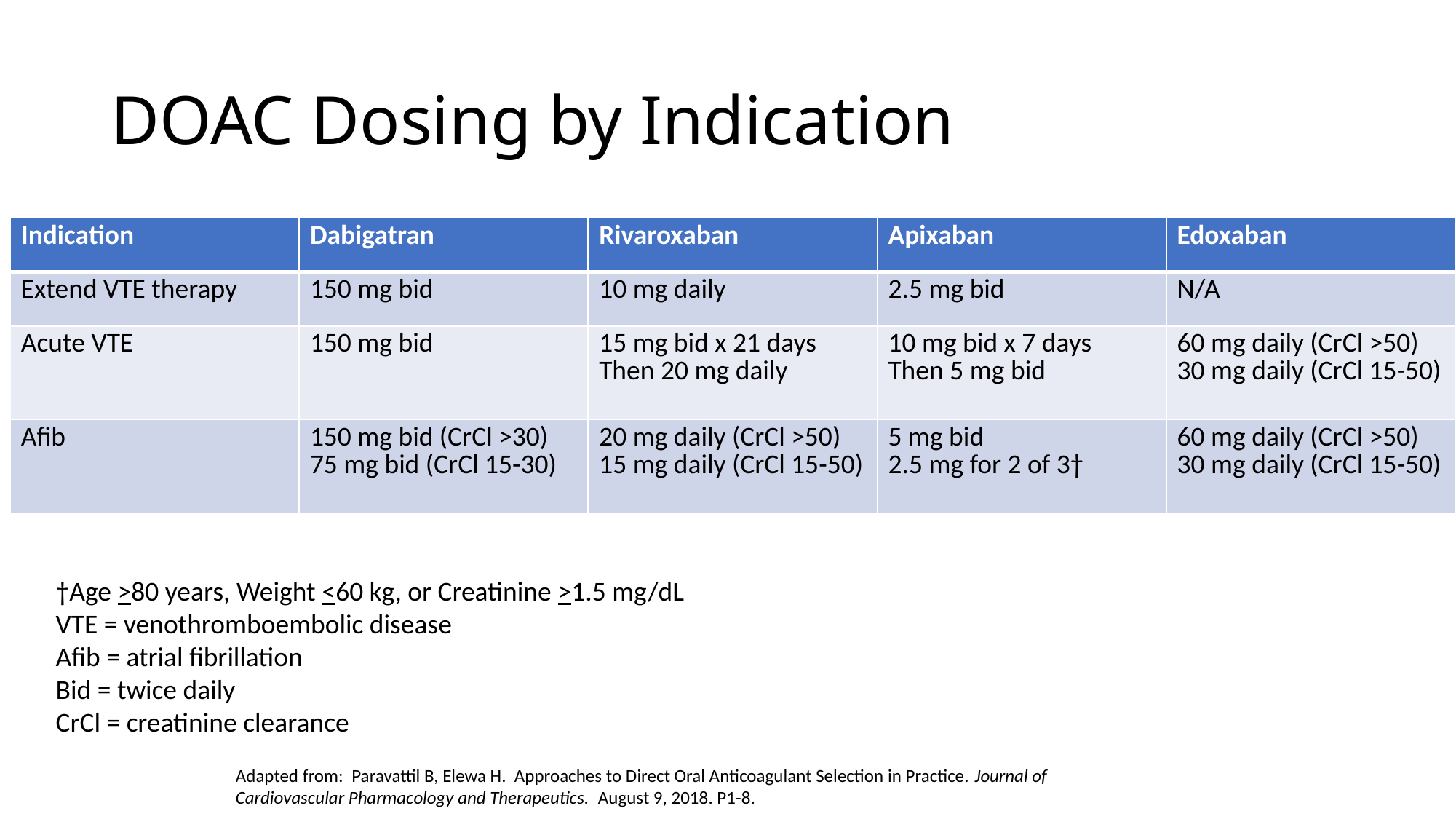

# DOAC Dosing by Indication
| Indication | Dabigatran | Rivaroxaban | Apixaban | Edoxaban |
| --- | --- | --- | --- | --- |
| Extend VTE therapy | 150 mg bid | 10 mg daily | 2.5 mg bid | N/A |
| Acute VTE | 150 mg bid | 15 mg bid x 21 days Then 20 mg daily | 10 mg bid x 7 days Then 5 mg bid | 60 mg daily (CrCl >50) 30 mg daily (CrCl 15-50) |
| Afib | 150 mg bid (CrCl >30) 75 mg bid (CrCl 15-30) | 20 mg daily (CrCl >50) 15 mg daily (CrCl 15-50) | 5 mg bid 2.5 mg for 2 of 3† | 60 mg daily (CrCl >50) 30 mg daily (CrCl 15-50) |
†Age >80 years, Weight <60 kg, or Creatinine >1.5 mg/dL
VTE = venothromboembolic disease
Afib = atrial fibrillation
Bid = twice daily
CrCl = creatinine clearance
Adapted from: Paravattil B, Elewa H. Approaches to Direct Oral Anticoagulant Selection in Practice. Journal of Cardiovascular Pharmacology and Therapeutics. August 9, 2018. P1-8.

## Slide 15
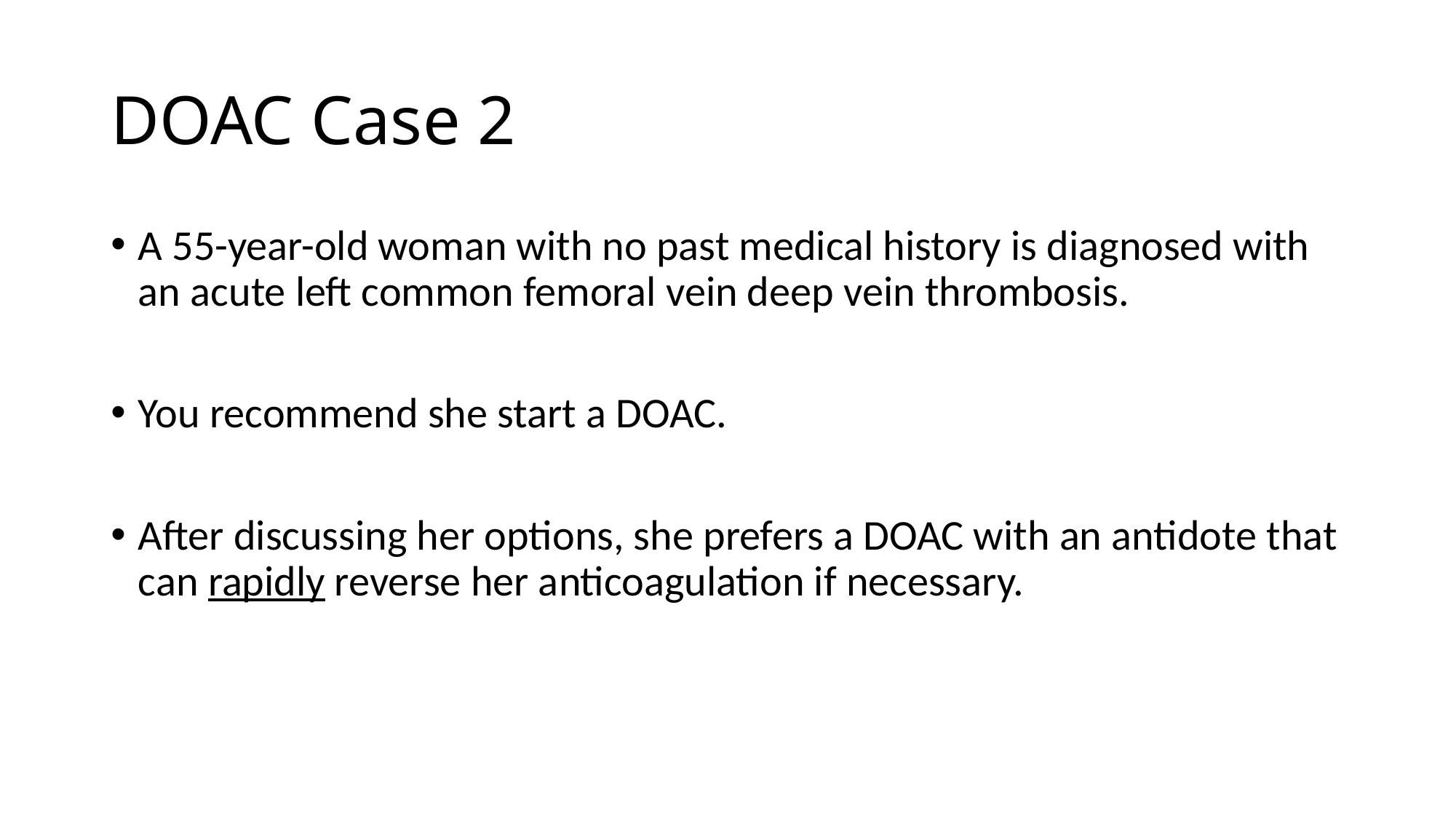

# DOAC Case 2
A 55-year-old woman with no past medical history is diagnosed with an acute left common femoral vein deep vein thrombosis.
You recommend she start a DOAC.
After discussing her options, she prefers a DOAC with an antidote that can rapidly reverse her anticoagulation if necessary.

## Slide 16
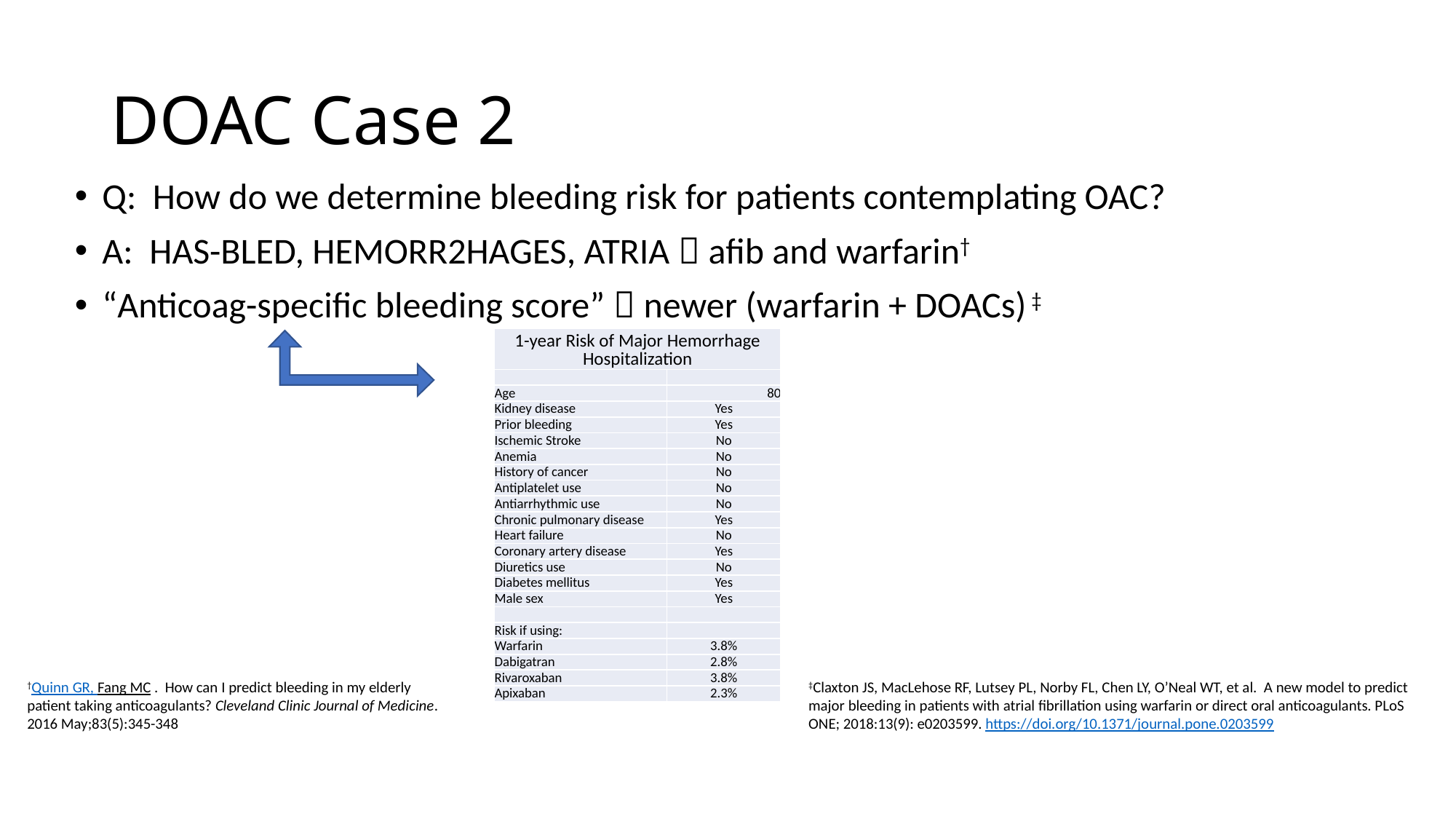

# DOAC Case 2
Q: How do we determine bleeding risk for patients contemplating OAC?
A: HAS-BLED, HEMORR2HAGES, ATRIA  afib and warfarin†
“Anticoag-specific bleeding score”  newer (warfarin + DOACs) ‡
| 1-year Risk of Major Hemorrhage Hospitalization | |
| --- | --- |
| | |
| Age | 80 |
| Kidney disease | Yes |
| Prior bleeding | Yes |
| Ischemic Stroke | No |
| Anemia | No |
| History of cancer | No |
| Antiplatelet use | No |
| Antiarrhythmic use | No |
| Chronic pulmonary disease | Yes |
| Heart failure | No |
| Coronary artery disease | Yes |
| Diuretics use | No |
| Diabetes mellitus | Yes |
| Male sex | Yes |
| | |
| Risk if using: | |
| Warfarin | 3.8% |
| Dabigatran | 2.8% |
| Rivaroxaban | 3.8% |
| Apixaban | 2.3% |
†Quinn GR, Fang MC . How can I predict bleeding in my elderly patient taking anticoagulants? Cleveland Clinic Journal of Medicine. 2016 May;83(5):345-348
‡Claxton JS, MacLehose RF, Lutsey PL, Norby FL, Chen LY, O’Neal WT, et al. A new model to predict major bleeding in patients with atrial fibrillation using warfarin or direct oral anticoagulants. PLoS ONE; 2018:13(9): e0203599. https://doi.org/10.1371/journal.pone.0203599

## Slide 17
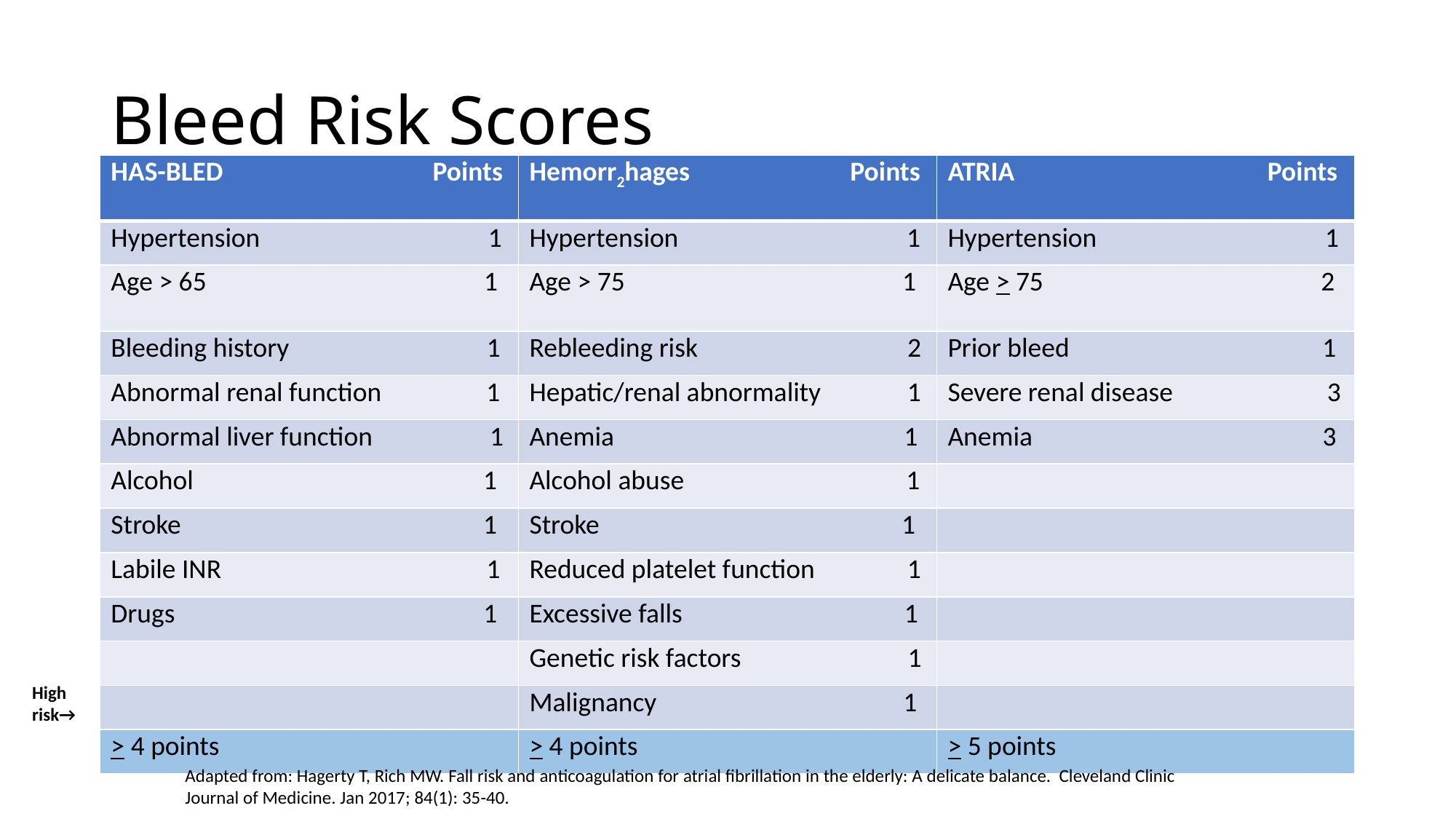

# Bleed Risk Scores
| HAS-BLED Points | Hemorr2hages Points | ATRIA Points |
| --- | --- | --- |
| Hypertension 1 | Hypertension 1 | Hypertension 1 |
| Age > 65 1 | Age > 75 1 | Age > 75 2 |
| Bleeding history 1 | Rebleeding risk 2 | Prior bleed 1 |
| Abnormal renal function 1 | Hepatic/renal abnormality 1 | Severe renal disease 3 |
| Abnormal liver function 1 | Anemia 1 | Anemia 3 |
| Alcohol 1 | Alcohol abuse 1 | |
| Stroke 1 | Stroke 1 | |
| Labile INR 1 | Reduced platelet function 1 | |
| Drugs 1 | Excessive falls 1 | |
| | Genetic risk factors 1 | |
| | Malignancy 1 | |
| > 4 points | > 4 points | > 5 points |
High risk→
Adapted from: Hagerty T, Rich MW. Fall risk and anticoagulation for atrial fibrillation in the elderly: A delicate balance. Cleveland Clinic Journal of Medicine. Jan 2017; 84(1): 35-40.

## Slide 18
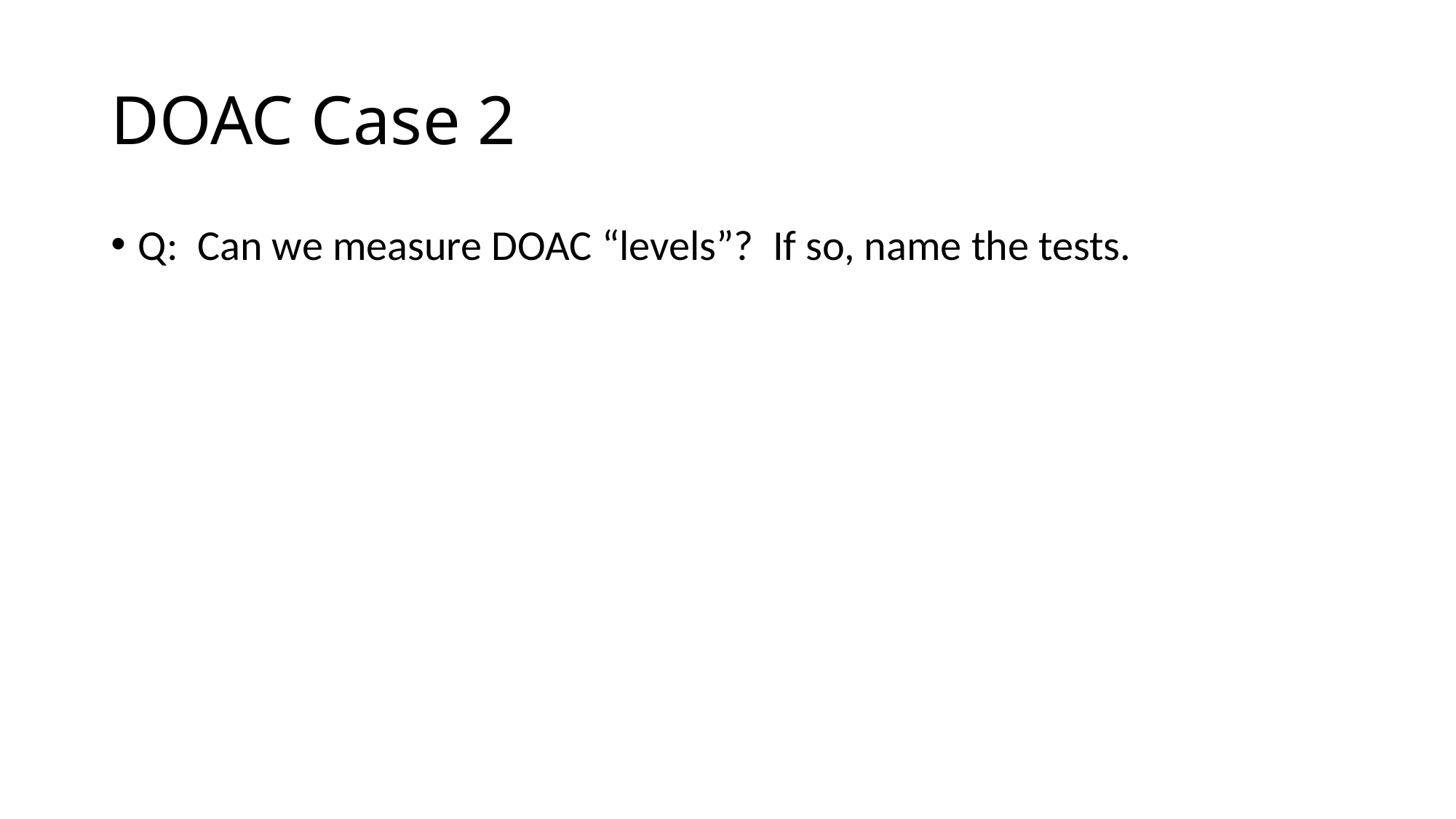

# DOAC Case 2
Q: Can we measure DOAC “levels”? If so, name the tests.

## Slide 19
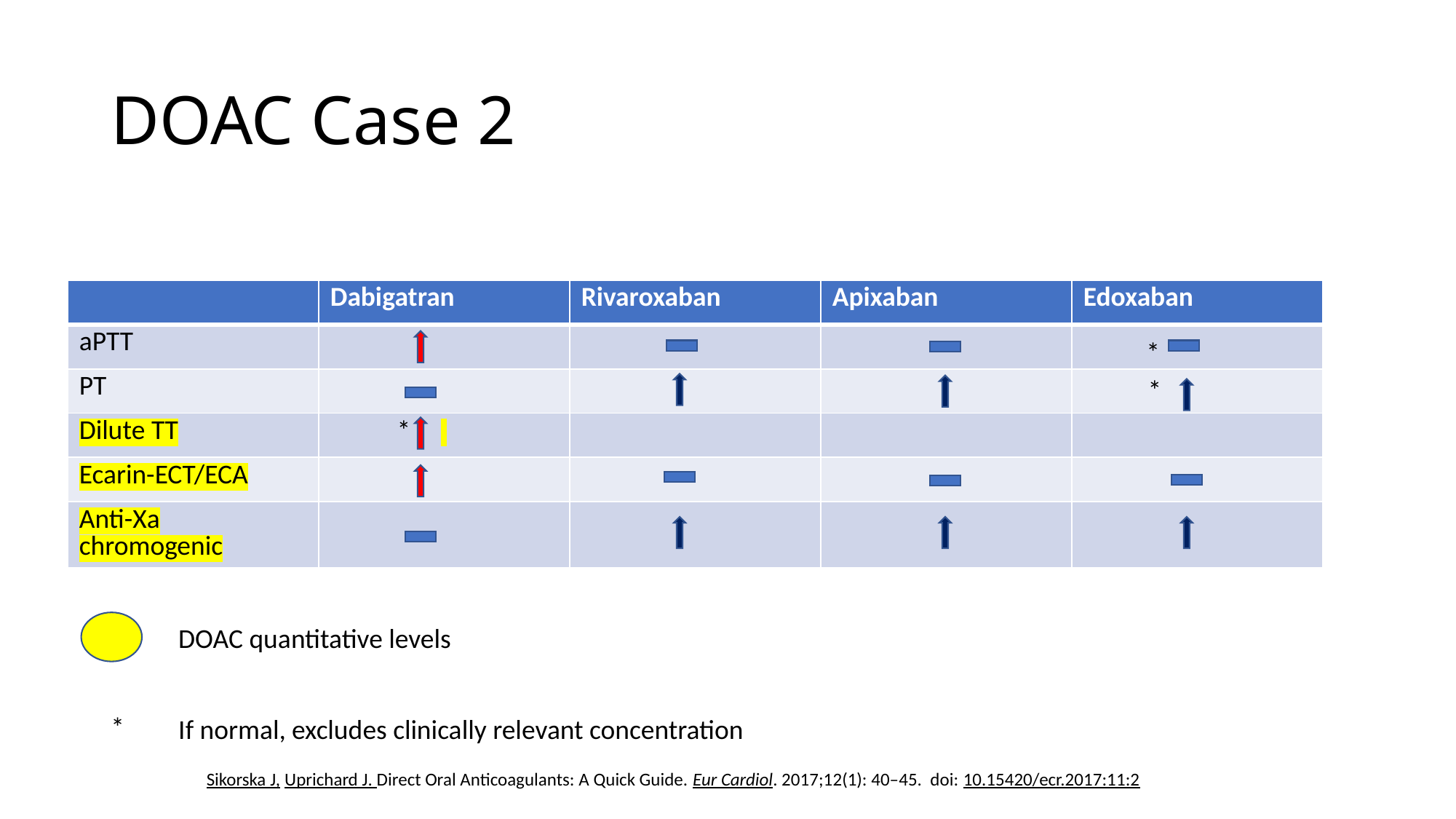

# DOAC Case 2
| | Dabigatran | Rivaroxaban | Apixaban | Edoxaban |
| --- | --- | --- | --- | --- |
| aPTT | | | | |
| PT | | | | |
| Dilute TT | | | | |
| Ecarin-ECT/ECA | | | | |
| Anti-Xa chromogenic | | | | |
*
*
*
DOAC quantitative levels
*
*
If normal, excludes clinically relevant concentration
Sikorska J, Uprichard J. Direct Oral Anticoagulants: A Quick Guide. Eur Cardiol. 2017;12(1): 40–45. doi: 10.15420/ecr.2017:11:2

## Slide 20
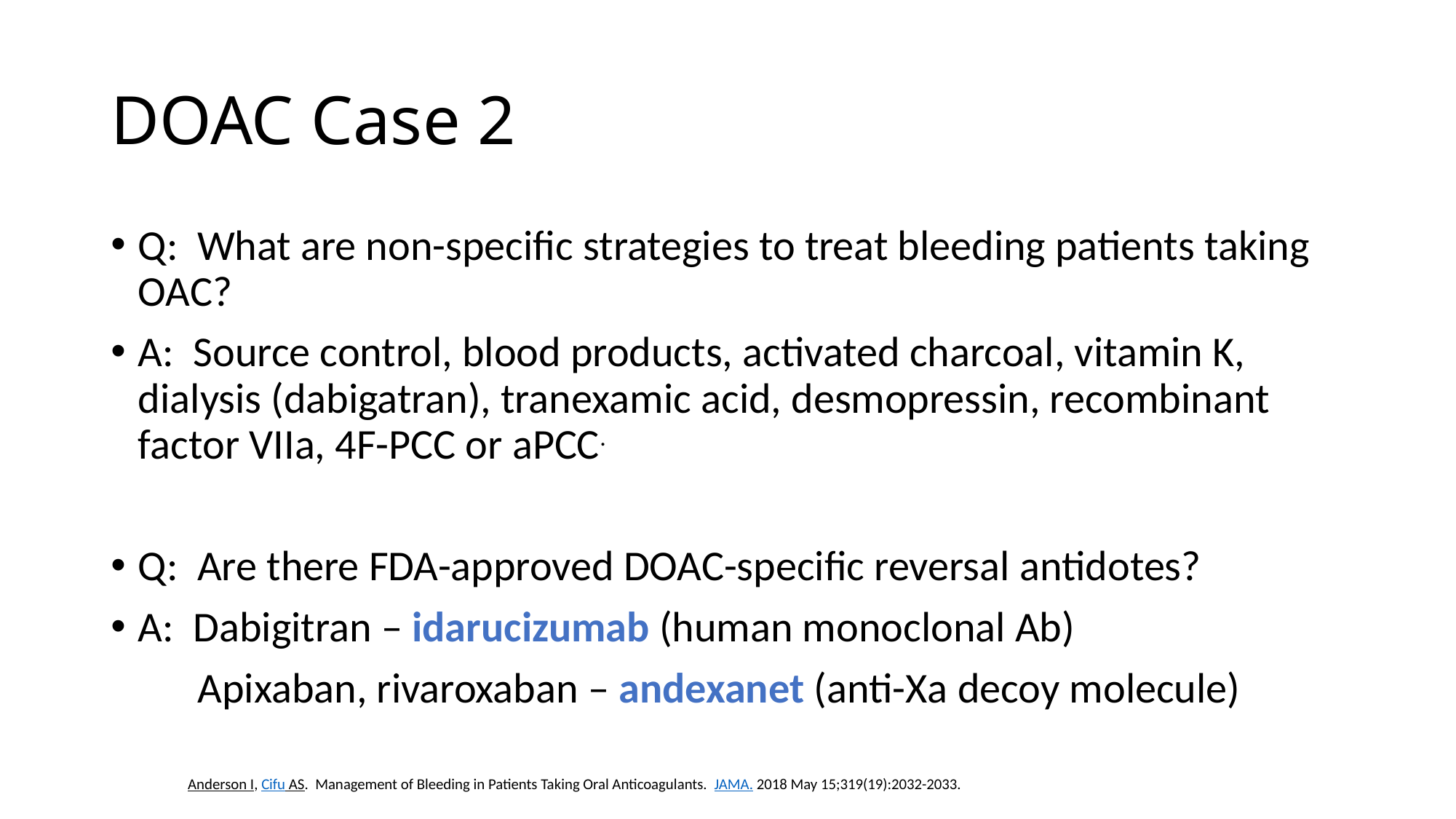

# DOAC Case 2
Q: What are non-specific strategies to treat bleeding patients taking OAC?
A: Source control, blood products, activated charcoal, vitamin K, dialysis (dabigatran), tranexamic acid, desmopressin, recombinant factor VIIa, 4F-PCC or aPCC.
Q: Are there FDA-approved DOAC-specific reversal antidotes?
A: Dabigitran – idarucizumab (human monoclonal Ab)
 Apixaban, rivaroxaban – andexanet (anti-Xa decoy molecule)
Anderson I, Cifu AS. Management of Bleeding in Patients Taking Oral Anticoagulants. JAMA. 2018 May 15;319(19):2032-2033.

## Slide 21
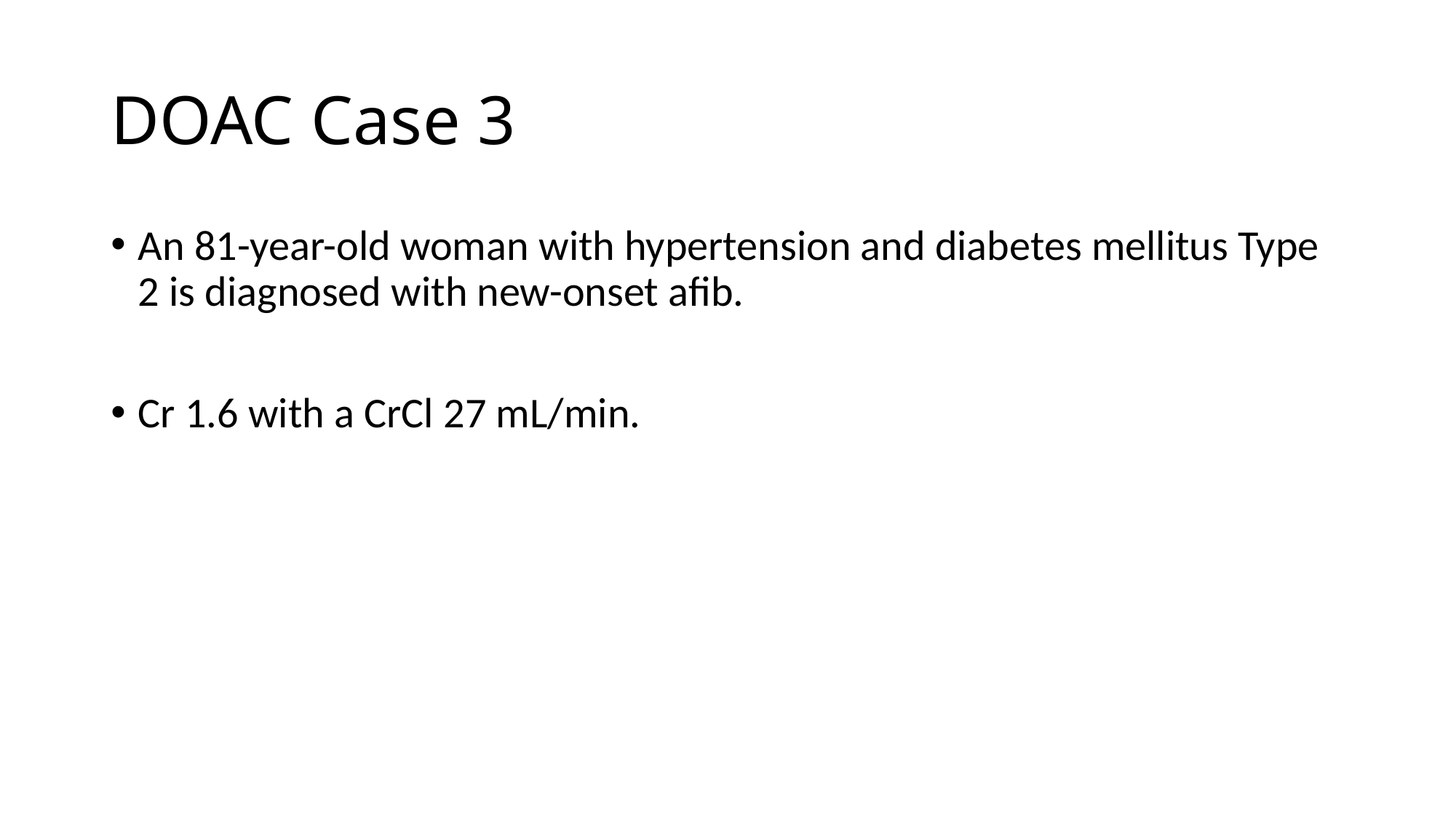

# DOAC Case 3
An 81-year-old woman with hypertension and diabetes mellitus Type 2 is diagnosed with new-onset afib.
Cr 1.6 with a CrCl 27 mL/min.

## Slide 22
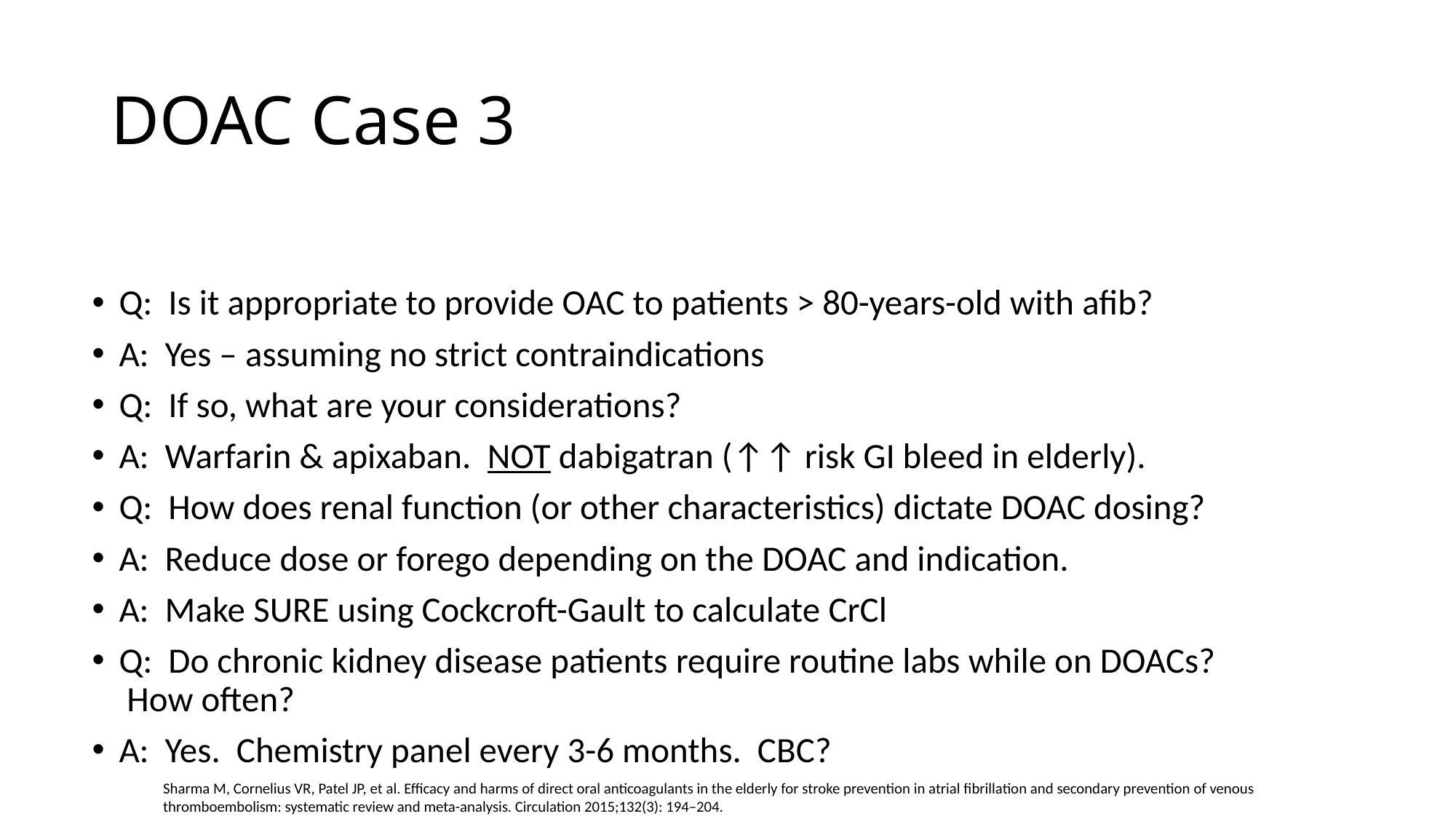

# DOAC Case 3
Q: Is it appropriate to provide OAC to patients > 80-years-old with afib?
A: Yes – assuming no strict contraindications
Q: If so, what are your considerations?
A: Warfarin & apixaban. NOT dabigatran (↑↑ risk GI bleed in elderly).
Q: How does renal function (or other characteristics) dictate DOAC dosing?
A: Reduce dose or forego depending on the DOAC and indication.
A: Make SURE using Cockcroft-Gault to calculate CrCl
Q: Do chronic kidney disease patients require routine labs while on DOACs? How often?
A: Yes. Chemistry panel every 3-6 months. CBC?
Sharma M, Cornelius VR, Patel JP, et al. Efficacy and harms of direct oral anticoagulants in the elderly for stroke prevention in atrial fibrillation and secondary prevention of venous thromboembolism: systematic review and meta-analysis. Circulation 2015;132(3): 194–204.

## Slide 23
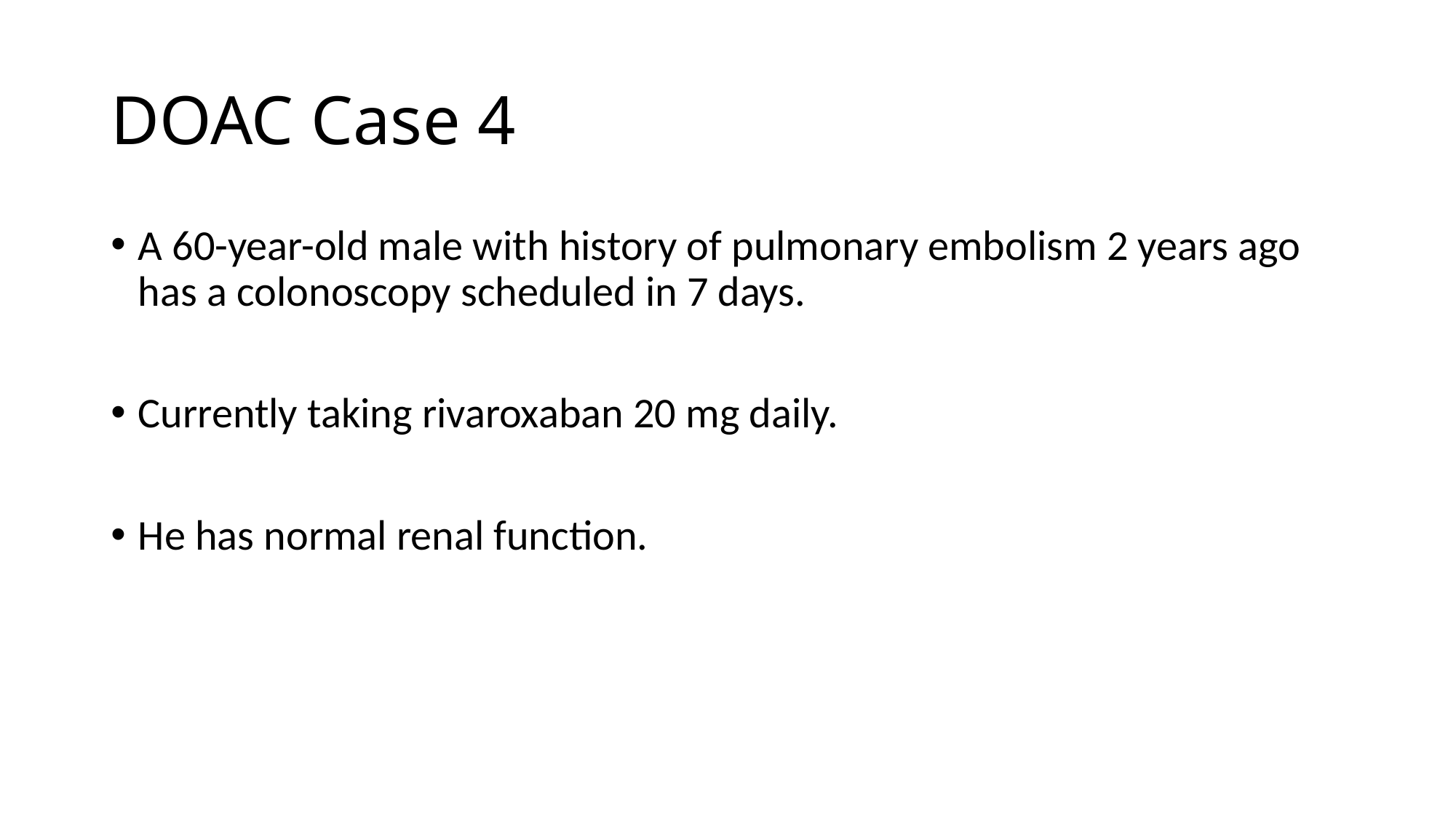

# DOAC Case 4
A 60-year-old male with history of pulmonary embolism 2 years ago has a colonoscopy scheduled in 7 days.
Currently taking rivaroxaban 20 mg daily.
He has normal renal function.

## Slide 24
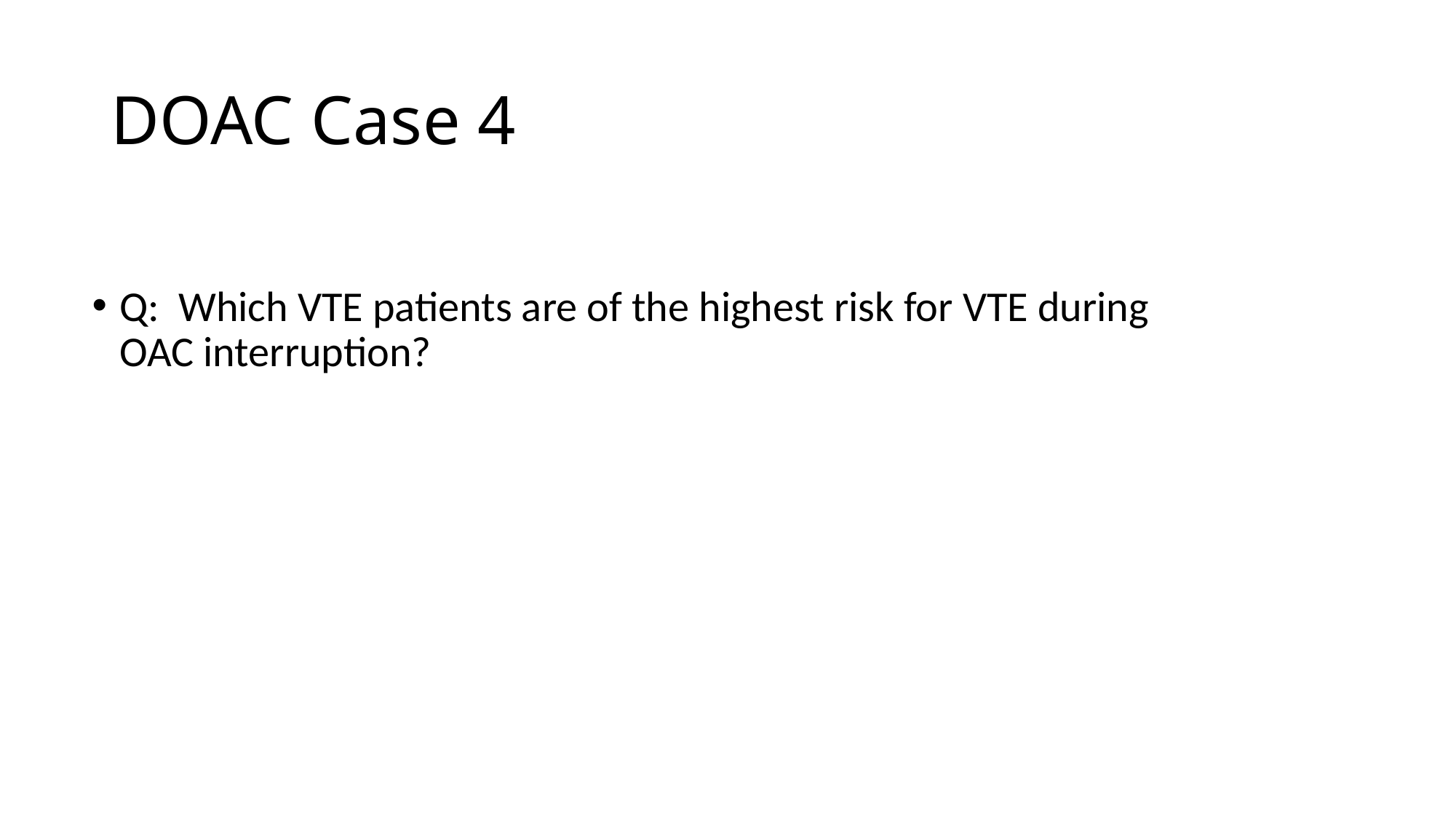

# DOAC Case 4
Q: Which VTE patients are of the highest risk for VTE during OAC interruption?

## Slide 25
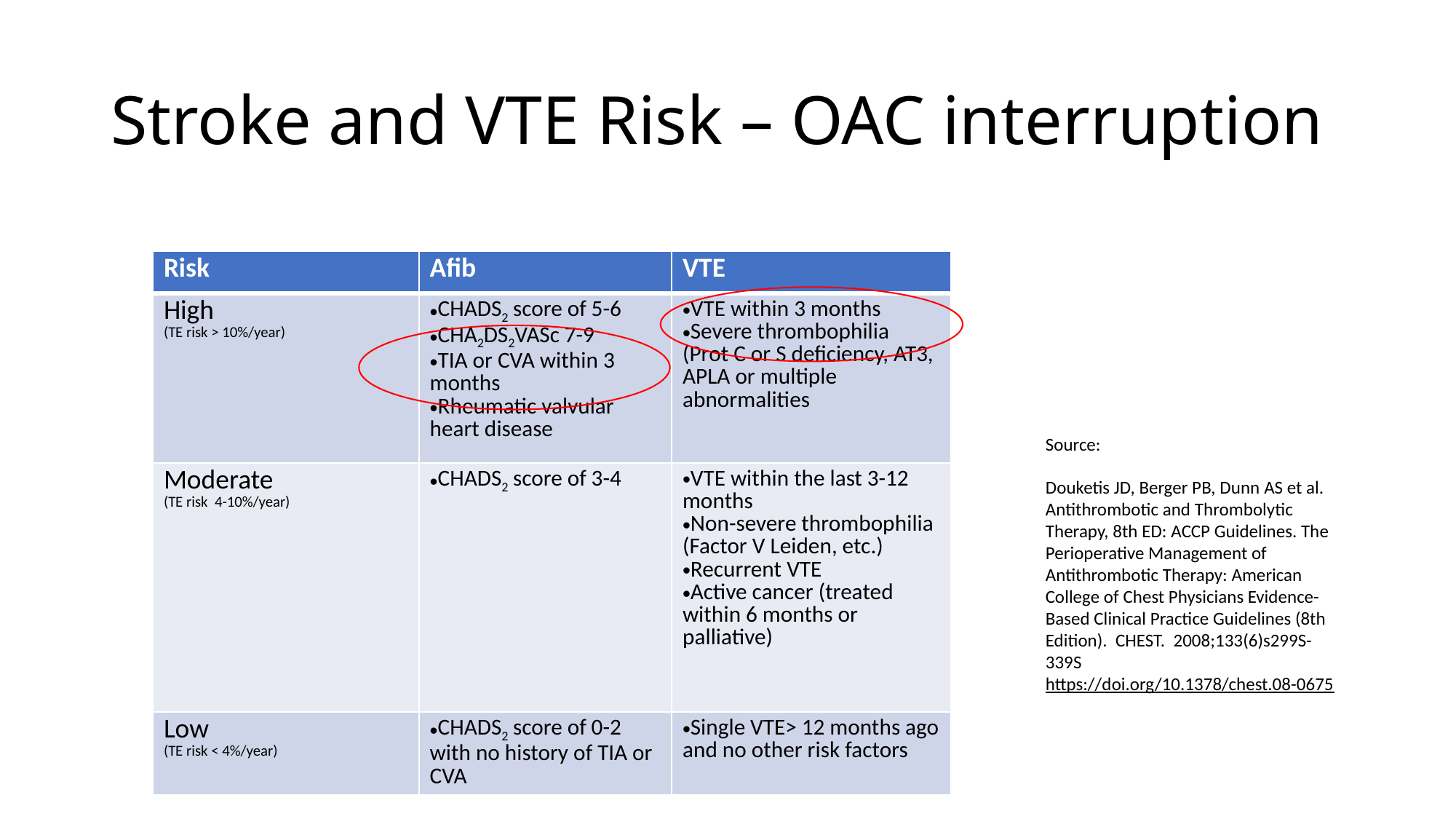

# Stroke and VTE Risk – OAC interruption
| Risk | Afib | VTE |
| --- | --- | --- |
| High (TE risk > 10%/year) | CHADS2 score of 5-6 CHA2DS2VASc 7-9 TIA or CVA within 3 months Rheumatic valvular heart disease | VTE within 3 months Severe thrombophilia (Prot C or S deficiency, AT3, APLA or multiple abnormalities |
| Moderate (TE risk 4-10%/year) | CHADS2 score of 3-4 | VTE within the last 3-12 months Non-severe thrombophilia (Factor V Leiden, etc.) Recurrent VTE Active cancer (treated within 6 months or palliative) |
| Low (TE risk < 4%/year) | CHADS2 score of 0-2 with no history of TIA or CVA | Single VTE> 12 months ago and no other risk factors |
Source:
Douketis JD, Berger PB, Dunn AS et al. Antithrombotic and Thrombolytic Therapy, 8th ED: ACCP Guidelines. The Perioperative Management of Antithrombotic Therapy: American College of Chest Physicians Evidence-Based Clinical Practice Guidelines (8th Edition). CHEST. 2008;133(6)s299S-339S https://doi.org/10.1378/chest.08-0675

## Slide 26
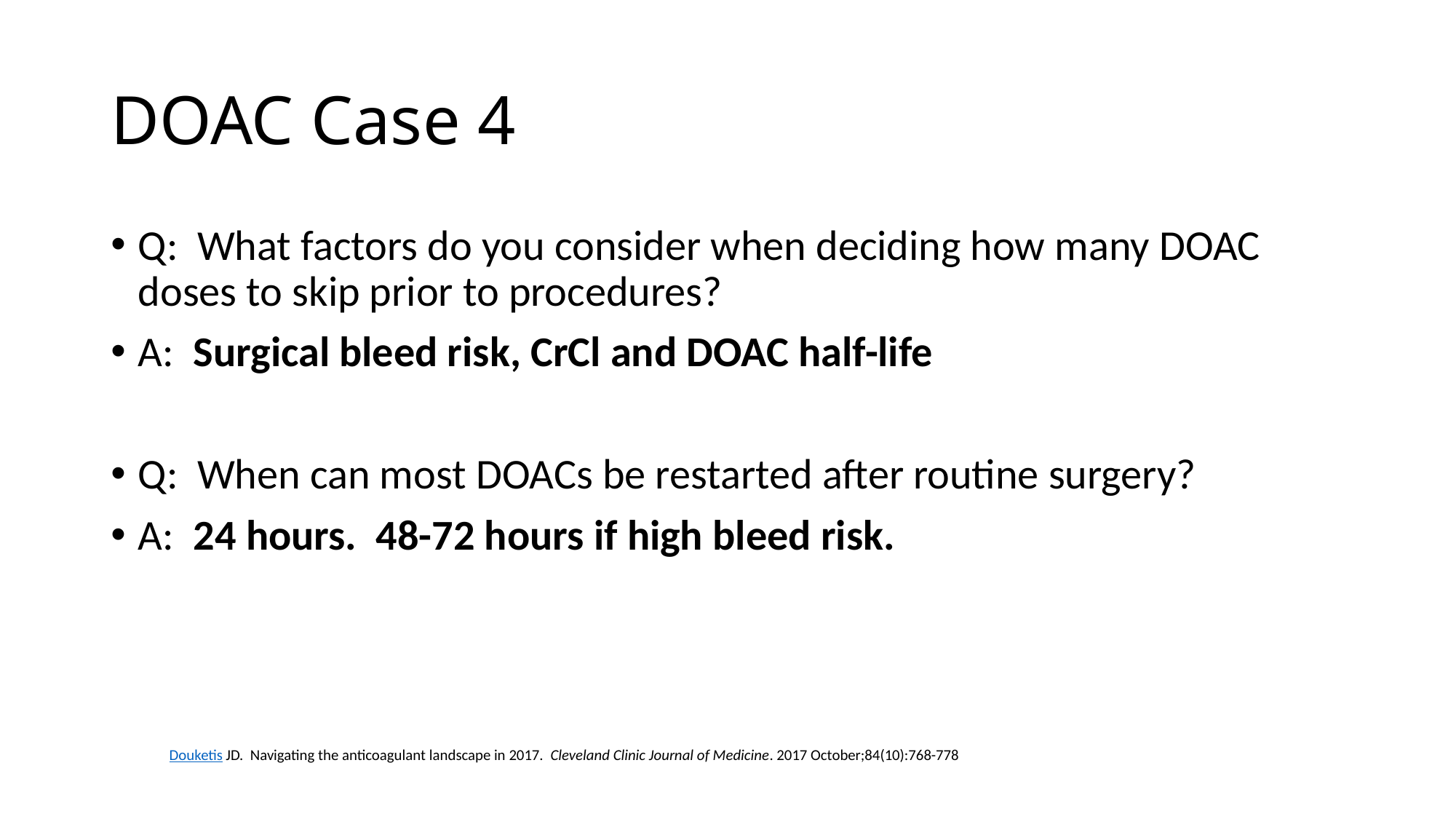

# DOAC Case 4
Q: What factors do you consider when deciding how many DOAC doses to skip prior to procedures?
A: Surgical bleed risk, CrCl and DOAC half-life
Q: When can most DOACs be restarted after routine surgery?
A: 24 hours. 48-72 hours if high bleed risk.
Douketis JD. Navigating the anticoagulant landscape in 2017. Cleveland Clinic Journal of Medicine. 2017 October;84(10):768-778

## Slide 27
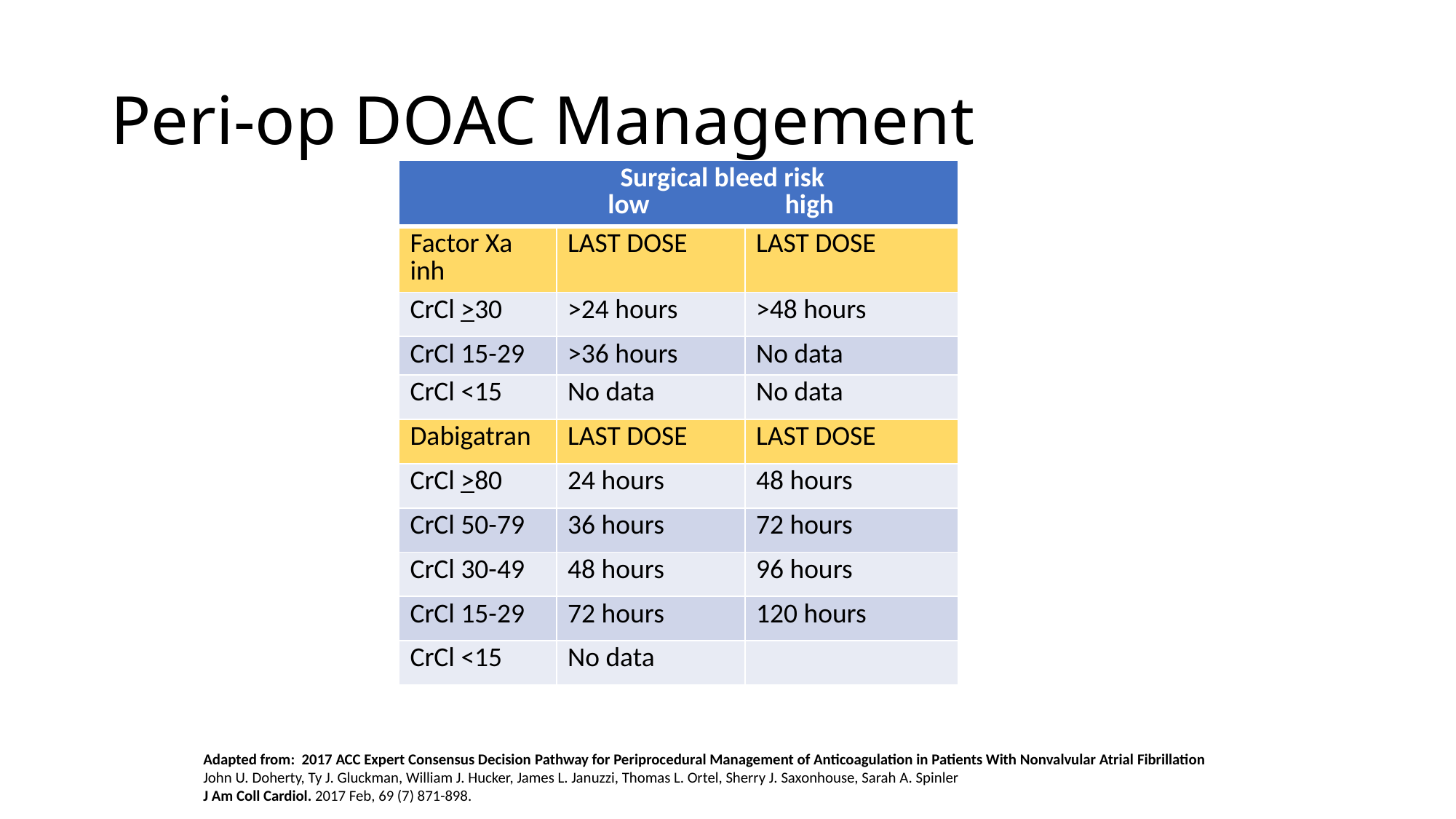

# Peri-op DOAC Management
| Surgical bleed risk low high | | |
| --- | --- | --- |
| Factor Xa inh | LAST DOSE | LAST DOSE |
| CrCl >30 | >24 hours | >48 hours |
| CrCl 15-29 | >36 hours | No data |
| CrCl <15 | No data | No data |
| Dabigatran | LAST DOSE | LAST DOSE |
| CrCl >80 | 24 hours | 48 hours |
| CrCl 50-79 | 36 hours | 72 hours |
| CrCl 30-49 | 48 hours | 96 hours |
| CrCl 15-29 | 72 hours | 120 hours |
| CrCl <15 | No data | |
Adapted from: 2017 ACC Expert Consensus Decision Pathway for Periprocedural Management of Anticoagulation in Patients With Nonvalvular Atrial Fibrillation
John U. Doherty, Ty J. Gluckman, William J. Hucker, James L. Januzzi, Thomas L. Ortel, Sherry J. Saxonhouse, Sarah A. Spinler
J Am Coll Cardiol. 2017 Feb, 69 (7) 871-898.

## Slide 28
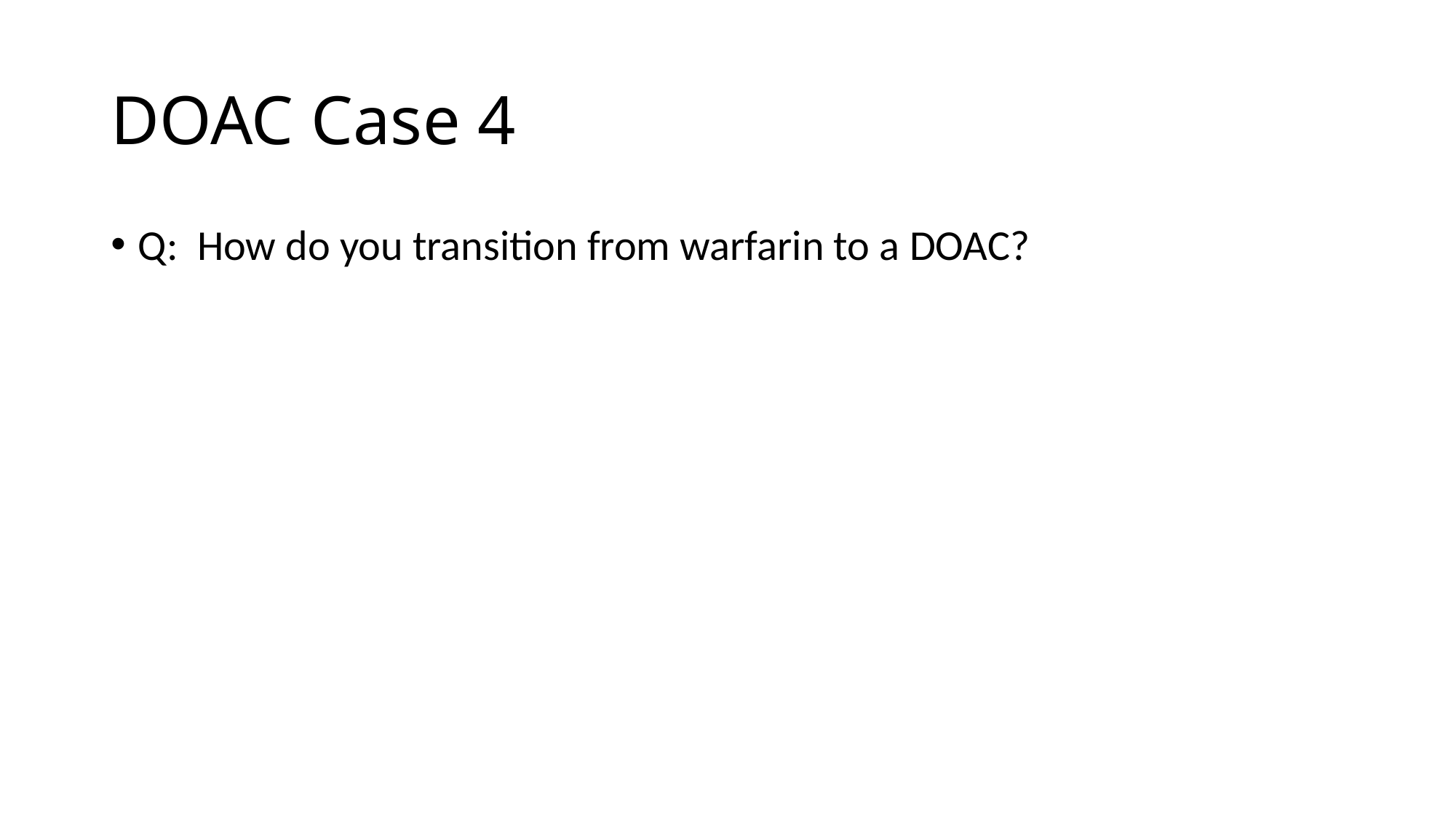

# DOAC Case 4
Q: How do you transition from warfarin to a DOAC?

## Slide 29
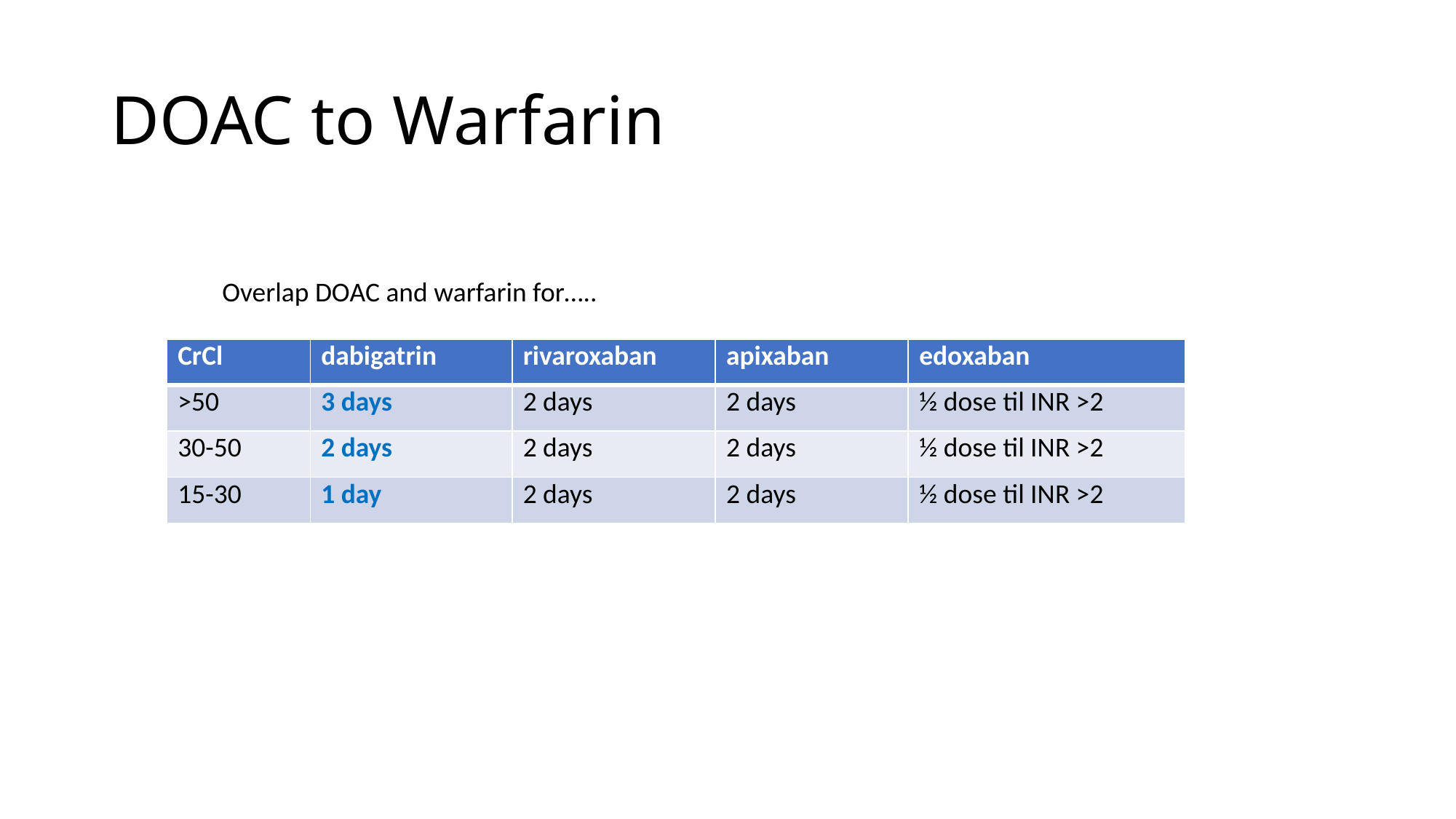

# DOAC to Warfarin
Overlap DOAC and warfarin for…..
| CrCl | dabigatrin | rivaroxaban | apixaban | edoxaban |
| --- | --- | --- | --- | --- |
| >50 | 3 days | 2 days | 2 days | ½ dose til INR >2 |
| 30-50 | 2 days | 2 days | 2 days | ½ dose til INR >2 |
| 15-30 | 1 day | 2 days | 2 days | ½ dose til INR >2 |

## Slide 30
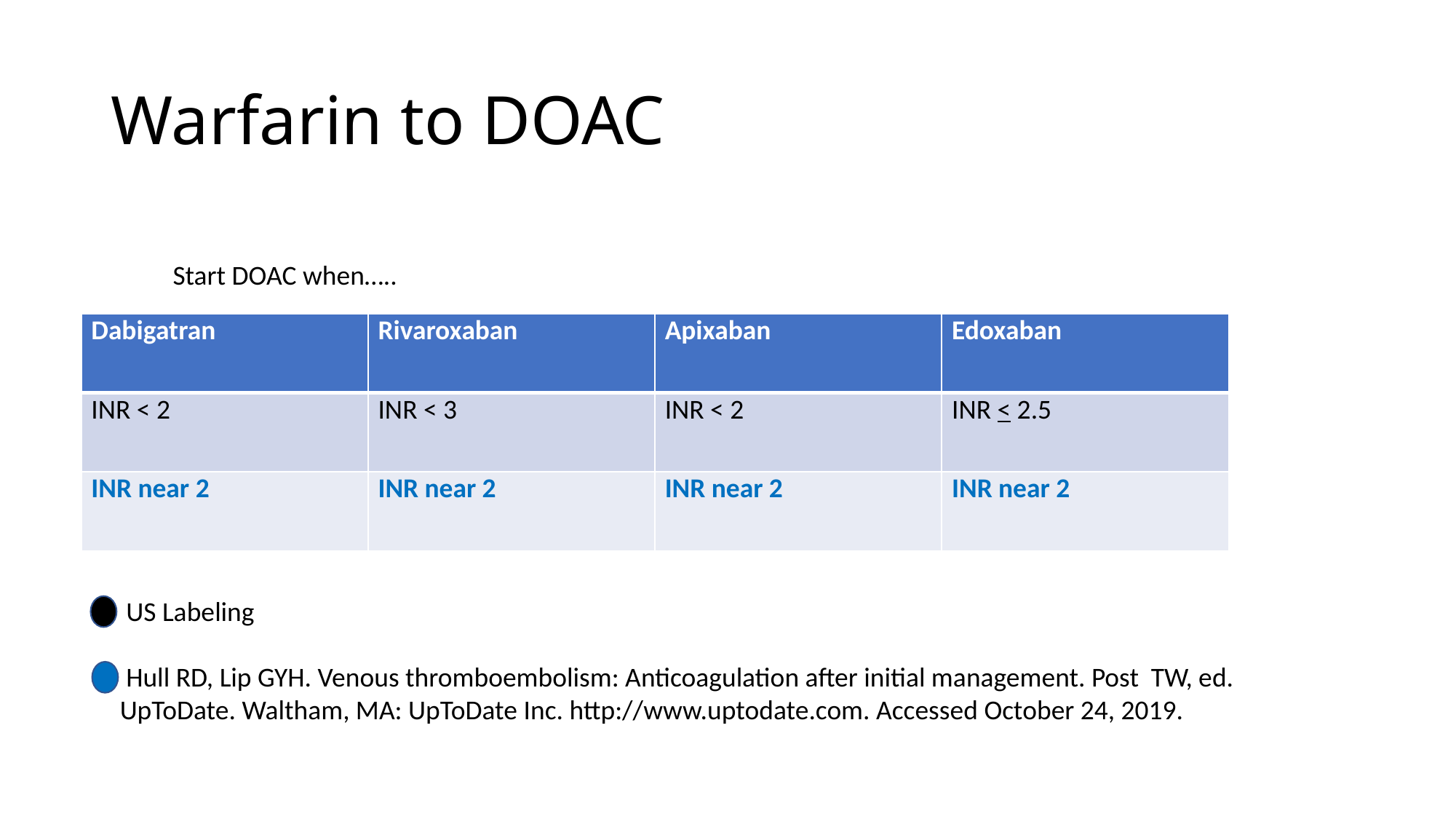

# Warfarin to DOAC
Start DOAC when…..
| Dabigatran | Rivaroxaban | Apixaban | Edoxaban |
| --- | --- | --- | --- |
| INR < 2 | INR < 3 | INR < 2 | INR < 2.5 |
| INR near 2 | INR near 2 | INR near 2 | INR near 2 |
 US Labeling
 Hull RD, Lip GYH. Venous thromboembolism: Anticoagulation after initial management. Post TW, ed. UpToDate. Waltham, MA: UpToDate Inc. http://www.uptodate.com. Accessed October 24, 2019.

## Slide 31
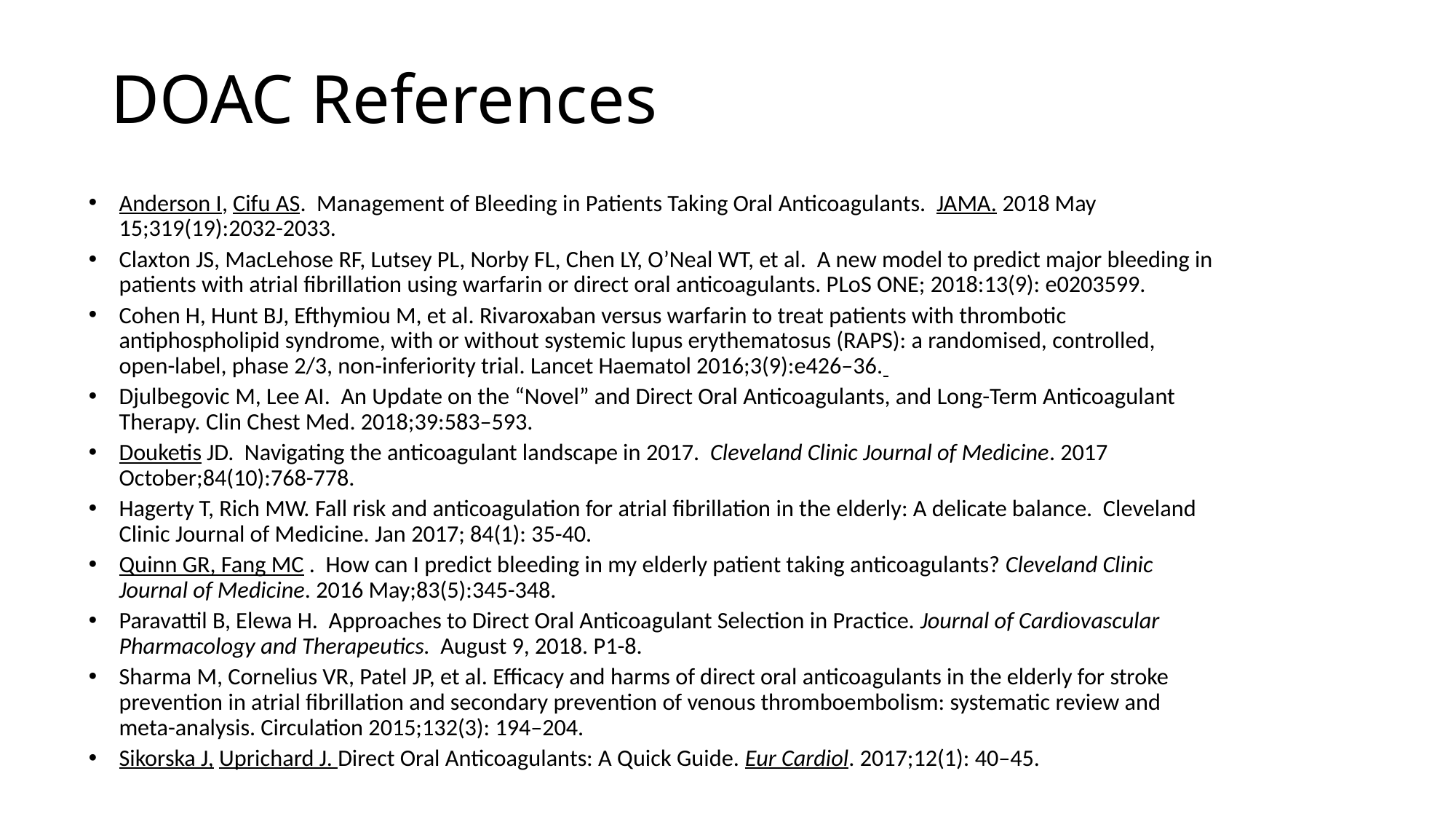

# DOAC References
Anderson I, Cifu AS. Management of Bleeding in Patients Taking Oral Anticoagulants. JAMA. 2018 May 15;319(19):2032-2033.
Claxton JS, MacLehose RF, Lutsey PL, Norby FL, Chen LY, O’Neal WT, et al. A new model to predict major bleeding in patients with atrial fibrillation using warfarin or direct oral anticoagulants. PLoS ONE; 2018:13(9): e0203599.
Cohen H, Hunt BJ, Efthymiou M, et al. Rivaroxaban versus warfarin to treat patients with thrombotic antiphospholipid syndrome, with or without systemic lupus erythematosus (RAPS): a randomised, controlled, open-label, phase 2/3, non-inferiority trial. Lancet Haematol 2016;3(9):e426–36.
Djulbegovic M, Lee AI. An Update on the “Novel” and Direct Oral Anticoagulants, and Long-Term Anticoagulant Therapy. Clin Chest Med. 2018;39:583–593.
Douketis JD. Navigating the anticoagulant landscape in 2017. Cleveland Clinic Journal of Medicine. 2017 October;84(10):768-778.
Hagerty T, Rich MW. Fall risk and anticoagulation for atrial fibrillation in the elderly: A delicate balance. Cleveland Clinic Journal of Medicine. Jan 2017; 84(1): 35-40.
Quinn GR, Fang MC . How can I predict bleeding in my elderly patient taking anticoagulants? Cleveland Clinic Journal of Medicine. 2016 May;83(5):345-348.
Paravattil B, Elewa H. Approaches to Direct Oral Anticoagulant Selection in Practice. Journal of Cardiovascular Pharmacology and Therapeutics. August 9, 2018. P1-8.
Sharma M, Cornelius VR, Patel JP, et al. Efficacy and harms of direct oral anticoagulants in the elderly for stroke prevention in atrial fibrillation and secondary prevention of venous thromboembolism: systematic review and meta-analysis. Circulation 2015;132(3): 194–204.
Sikorska J, Uprichard J. Direct Oral Anticoagulants: A Quick Guide. Eur Cardiol. 2017;12(1): 40–45.
