# Supplementary material for: Direct-Acting Oral Anticoagulants: A Resident-Based Workshop to Improve Knowledge and Confidence
Source: MedEdPORTAL. 2020 Sep 30;16:10981. doi: 10.15766/mep_2374-8265.10981 (PMC7526504; doi:10.15766/mep_2374-8265.10981)
Supplement: Supplementary file 1 — Preworkshop MCQ Students.docxDOAC PowerPoint.pptDOAC Indications and Dosing Case.docxDOAC Monitoring and Reversal Case.docxDOAC Dosing Elderly Case.docxDOAC Peri-procedural Case.docxPostworkshop MCQ and Confidence Survey Students.docxPostworkshop MCQ Facilitators.docx [file mep_2374-8265.10981-s001.zip › C. DOAC Indications and Dosing Case.docx]

**Learner Case 1.**

A 77-year-old male with hypertension presents to the Emergency Department with dyspnea and bilateral leg edema. Blood pressure 155/85, heart rate 115 beats per minute. Creatinine clearance is 55 mL/min. EKG reveals atrial fibrillation (afib). An echocardiogram shows a normal ejection fraction with grade 1 diastolic dysfunction with normal valve function.

Learning/discussion questions:

1. How do we calculate annual stroke risk in afib and who requires oral anticoagulation (OAC)?
2. What considerations do you weigh when choosing DOAC/warfarin?
3. Name some DOAC cautions/contraindications.
4. How does renal function factor in to DOAC dosing?
