# Supplementary material for: Direct-Acting Oral Anticoagulants: A Resident-Based Workshop to Improve Knowledge and Confidence
Source: MedEdPORTAL. 2020 Sep 30;16:10981. doi: 10.15766/mep_2374-8265.10981 (PMC7526504; doi:10.15766/mep_2374-8265.10981)
Supplement: Supplementary file 1 — Preworkshop MCQ Students.docxDOAC PowerPoint.pptDOAC Indications and Dosing Case.docxDOAC Monitoring and Reversal Case.docxDOAC Dosing Elderly Case.docxDOAC Peri-procedural Case.docxPostworkshop MCQ and Confidence Survey Students.docxPostworkshop MCQ Facilitators.docx [file mep_2374-8265.10981-s001.zip › D. DOAC Monitoring and Reversal Case.docx]

**Learner Case 2.**

A 55-year-old woman with no past medical history is diagnosed with an acute left common femoral vein deep venous thrombosis. You recommend she start a DOAC. After discussing her options, she prefers a DOAC with an antidote that can rapidly reverse her anticoagulation if necessary.

Learning/discussion questions:

1. How do we determine bleeding risk for patients contemplating OAC?
2. Can we measure DOAC “levels”? If so, name the tests.
3. What are non-specific strategies to treat bleeding patients taking OAC?
4. What do you know about DOAC-specific reversal antidotes?
