# Supplementary material for: Direct-Acting Oral Anticoagulants: A Resident-Based Workshop to Improve Knowledge and Confidence
Source: MedEdPORTAL. 2020 Sep 30;16:10981. doi: 10.15766/mep_2374-8265.10981 (PMC7526504; doi:10.15766/mep_2374-8265.10981)
Supplement: Supplementary file 1 — Preworkshop MCQ Students.docxDOAC PowerPoint.pptDOAC Indications and Dosing Case.docxDOAC Monitoring and Reversal Case.docxDOAC Dosing Elderly Case.docxDOAC Peri-procedural Case.docxPostworkshop MCQ and Confidence Survey Students.docxPostworkshop MCQ Facilitators.docx [file mep_2374-8265.10981-s001.zip › E. DOAC Dosing Elderly Case.docx]

**Learner Case 3.**

An 81-year-old woman with hypertension and diabetes is diagnosed with new-onset atrial fibrillation. Her creatinine is 1.6 with a creatinine clearance of 27 mL/min.

Learning/discussion questions:

1. Is it appropriate to provide OAC to patients > 80 yo with afib?
2. If so, what are your considerations?
3. How does renal function (or other characteristics) dictate DOAC dosing?
4. Do CKD patients require routine labs while on DOAC? How often?
