# Supplementary material for: Direct-Acting Oral Anticoagulants: A Resident-Based Workshop to Improve Knowledge and Confidence
Source: MedEdPORTAL. 2020 Sep 30;16:10981. doi: 10.15766/mep_2374-8265.10981 (PMC7526504; doi:10.15766/mep_2374-8265.10981)
Supplement: Supplementary file 1 — Preworkshop MCQ Students.docxDOAC PowerPoint.pptDOAC Indications and Dosing Case.docxDOAC Monitoring and Reversal Case.docxDOAC Dosing Elderly Case.docxDOAC Peri-procedural Case.docxPostworkshop MCQ and Confidence Survey Students.docxPostworkshop MCQ Facilitators.docx [file mep_2374-8265.10981-s001.zip › F. DOAC Peri-Procedural Case.docx]

**Learner Case 4.**

A 60-year-old male with a history of pulmonary embolism 2 years ago has a colonoscopy scheduled in 7 days. He is currently taking rivaroxaban 20 mg daily. He has normal renal function.

Learning/discussion questions:

1. Which VTE patients are of the highest risk for VTE during OAC interruption?
2. What factors do you consider when deciding how many DOAC doses to skip?
3. When can most DOACs be restarted after routine surgery?
4. Your patient wants to switch to warfarin due to cost. How do you transition?
