# Supplementary material for: Direct-Acting Oral Anticoagulants: A Resident-Based Workshop to Improve Knowledge and Confidence
Source: MedEdPORTAL. 2020 Sep 30;16:10981. doi: 10.15766/mep_2374-8265.10981 (PMC7526504; doi:10.15766/mep_2374-8265.10981)
Supplement: Supplementary file 1 — Preworkshop MCQ Students.docxDOAC PowerPoint.pptDOAC Indications and Dosing Case.docxDOAC Monitoring and Reversal Case.docxDOAC Dosing Elderly Case.docxDOAC Peri-procedural Case.docxPostworkshop MCQ and Confidence Survey Students.docxPostworkshop MCQ Facilitators.docx [file mep_2374-8265.10981-s001.zip › H. Postworkshop MCQ Facilitators.docx]

**IM Resident Direct-Acting Oral Anticoagulation (DOAC) Survey: Facilitator Guide**

**Thank you for participating in this survey. I am assessing residents’ knowledge around and comfort prescribing the DOACs dabigatran, rivaroxaban, apixaban and edoxaban. Your input on this survey is greatly appreciated!**

**Date:**

**PGY-1 PGY-2 PGY-3**

**Have you rotated through the Anticoagulation Management Service (AMS) Clinic?**

**Yes No**

**Answer the below questions to the best of your ability:**

**1.** A 77-year-old male with hypertension presents to the Emergency Department with dyspnea and bilateral leg edema. Blood pressure 155/85, heart rate 115 beats per minute. Creatinine clearance is 55 mL/min. EKG reveals atrial fibrillation. An echocardiogram shows a normal ejection fraction with grade 1 diastolic dysfunction with normal valve function.

Which of the following statements is most correct?Top of Form

A This patient does not require oral anticoagulation

B Start enoxaparin 1 mg/kg subcutaneous every 12 hours + warfarin 5 mg qhs

**C Start rivaroxaban 20 mg every evening**

D Admit for heparin intravenous drip and warfarin initiation

*Facilitator Notes:*

1. CHA2DS2VASc score = 4. Requires anticoagulation.
2. Rivaroxaban better option with no obvious contraindications.

(d) Rivaroxaban better option with no obvious contraindications.

**2**. A 55-year-old woman with no past medical history is diagnosed with an acute left common femoral vein deep venous thrombosis. You recommend she start a DOAC. After discussing her options, she prefers a DOAC with an antidote that can rapidly reverse her anticoagulation if necessary.

Which of the following DOACs does NOT have an FDA-approved anticoagulation reversal antidote?Top of Form

A apixaban

B rivaroxaban

**C edoxaban**

D dabigatran

*Facilitator Notes:*

Apixaban and rivaroxaban reversal agent = andexanet

Dabigatran reversal agent = idarucizumab

**3**. An 81-year-old woman with hypertension and diabetes is diagnosed with new-onset atrial fibrillation. Her creatinine is 1.6 with a creatinine clearance of 27 mL/min.

Which of the following DOACs agents and dosing would you recommend?

A rivaroxaban 10 mg daily

**B apixaban 2.5 mg bid**

C dabigatran 110 mg bid

D edoxaban 60 mg daily

*Facilitator Notes:*

Rivaroxaban dose should be 15 mg for CrCl 15-50 mL/

Dabigatran dose should be 75 mg bid for for CrCl 15-30 mL/min

Edoxaban dose should be 30 mg for CrCl 15-50 mL/min

**4.** A 60-year-old male with a history of pulmonary embolism 2 years ago has a colonoscopy scheduled in 7 days. He is currently taking rivaroxaban 20 mg daily. He has normal renal function.

What is the best approach to his peri-procedural anticoagulation management?

A Stop the rivaroxaban now

B Stop the rivaroxaban now and start enoxaparin 1 mg/kg sq every 12 hours

C Skip 3 doses of the rivaroxaban prior to colonoscopy

**D Skip 1 dose of rivaroxaban prior to colonoscopy**

*Facilitator Notes:*

Skip 1 dose for low bleed risk procedure, normal renal function and low acute VTE risk with OAC interruption

| **Please Rate Your LEVEL OF CONFIDENCE with the Following Before the Workshop** | | | | | |
| --- | --- | --- | --- | --- | --- |
|  | Not confident at all | Minimally confident | Somewhat confident | Reasonably confident | Highly confident |
| Weighing the pros and cons of the various DOAC options | 1 | 2 | 3 | 4 | 5 |
| Choosing the appropriate initial dose for DOACs based on indication/comorbidities | 1 | 2 | 3 | 4 | 5 |
| Converting to/from warfarin/DOACs | 1 | 2 | 3 | 4 | 5 |
| DOAC contraindications and adverse effects | 1 | 2 | 3 | 4 | 5 |
| DOAC cost and insurance coverage | 1 | 2 | 3 | 4 | 5 |

| **Please Rate Your LEVEL OF CONFIDENCE with the Following After the Workshop** | | | | | |
| --- | --- | --- | --- | --- | --- |
|  | Not confident at all | Minimally confident | Somewhat confident | Reasonably confident | Highly confident |
| Weighing the pros and cons of the various DOAC options | 1 | 2 | 3 | 4 | 5 |
| Choosing the appropriate initial dose for DOACs based on indication/comorbidities | 1 | 2 | 3 | 4 | 5 |
| Converting to/from warfarin/DOACs | 1 | 2 | 3 | 4 | 5 |
| DOAC contraindications and adverse effects | 1 | 2 | 3 | 4 | 5 |
| DOAC cost and insurance coverage | 1 | 2 | 3 | 4 | 5 |

| **Please Rate Your LEVEL OF SATISFACTION with the Following** | | | | | |
| --- | --- | --- | --- | --- | --- |
|  | Strongly disagree | Disagree | Neither agree or disagree | Agree | Strongly agree |
| I am satisfied with the teaching that I have received regarding DOACs | 1 | 2 | 3 | 4 | 5 |

Would you be interested in additional DOAC-specific training? Yes No

In this space, please let us know if any other thoughts, questions, concerns around DOACs or anticoagulation.
