# Supplementary material for: Direct-Acting Oral Anticoagulants: A Resident-Based Workshop to Improve Knowledge and Confidence
Source: MedEdPORTAL. 2020 Sep 30;16:10981. doi: 10.15766/mep_2374-8265.10981 (PMC7526504; doi:10.15766/mep_2374-8265.10981)
Supplement: Supplementary file 1 — Preworkshop MCQ Students.docxDOAC PowerPoint.pptDOAC Indications and Dosing Case.docxDOAC Monitoring and Reversal Case.docxDOAC Dosing Elderly Case.docxDOAC Peri-procedural Case.docxPostworkshop MCQ and Confidence Survey Students.docxPostworkshop MCQ Facilitators.docx [file mep_2374-8265.10981-s001.zip › _Educational_Summary_Report_10981.pdf]

MedEdPORTAL® The AAMC Journal of Teaching and Learning Resources

Original Publication Open Access

Direct-Acting Oral Anticoagulants: A Resident-Based Workshop to Improve Knowledge and Confidence

Irsk Anderson, MD*, Vineet M. Arora, MD

*Corresponding Author: ianderso@medicine.bsd.uchicago.edu

Abstract

Introduction: Direct-acting oral anticoagulant (DOAC) prescriptions have increased steadily since the first, dabigatran, was Food and Drug Administration-approved in 2010. They have multiple advantages over vitamin K antagonists including fixed dosing without coagulation lab monitoring, rapid onset and offset of action, and fewer drug and food interactions. Patient-specific dosing, administration education, adherence, and monitoring are critically important. Many providers are unfamiliar with these concepts and too often use DOACs for off-label indications or at off-label dosing. A DOAC workshop was created to address knowledge gaps and improve internal medicine resident prescribing confidence. **Methods:** One author (Irsk Anderson) conducted four 1-hour DOAC workshops with 49 total internal medicine residents rotating on their outpatient clinical rotation between October 2018 and November 2019. Residents performed small-group learning around four DOAC-specific cases, followed by a large-group report-out session. The residents completed pre- and postworkshop multiple-choice questions (MCQs) to assess knowledge as well as a postworkshop DOAC confidence self-assessment. **Results:** Resident knowledge, assessed by percentage of residents answering correctly, improved significantly for all four MCQs after completing the workshop (all $p < .003$). Resident confidence, assessed on a 5-point Likert scale, improved significantly for all five themes ($p < .001$). Overall resident satisfaction was high ($M = 4.2$ on a 5-point Likert scale) and 85% desired further DOAC training. **Discussion:** A 1-hour DOAC workshop was feasible and improved internal medicine resident knowledge and self-confidence. Future work should assess behavior change and patient clinical outcomes.

Keywords
Direct-Acting Anticoagulants, Medical Residents, Physician, Internal Medicine, Case-Based Learning

Educational Objectives

By the end of this activity, learners will be able to:

1. Name direct-acting oral anticoagulants (DOACs) indications, contraindications, and cautions.
2. Differentiate DOAC dosing by indication and comorbidities.
3. Identify DOAC lab monitoring tests and reversal agents.
4. Practice DOAC peri-procedural management.

Introduction

The first direct-acting oral anticoagulant (DOAC), dabigatran, was Food and Drug Administration (FDA)-approved for use in 2010. Since then, four additional DOACs (rivaroxaban, apixaban, edoxaban, betrixaban) have been FDA-approved. DOAC prescriptions for atrial fibrillation currently outnumber those for warfarin in North America and Europe.[1] Multiple specialty organizations have released guidelines preferring DOACs as first-line oral anticoagulation or a suitable alternative to vitamin K antagonists for multiple indications, including the prevention and treatment of venothromboembolic disease as well as for stroke prevention in nonvalvular atrial fibrillation.[2-4] Multiple studies have shown that DOACs are frequently used off-label and at incorrect doses.[5,6] Such practice places patients at risk for bleeding and thromboembolic events.[7,8] Inappropriate prescribing is likely a consequence of inadequate DOAC education, familiarity, and prescribing experience. Piran et al. published two survey-based studies looking at health care provider knowledge and comfort with DOACs.[9,10] The first study, a single institution-based study consisting of 48% resident physicians and 40% staff physicians from four separate specialties (hematology, thrombosis medicine, general

Citation:
Anderson I, Arora VM. Direct-acting oral anticoagulants: a resident-based workshop to improve knowledge and confidence. *MedEdPORTAL*. 2020;16:10981.
https://doi.org/10.15766/mep_2374-8265.10981

Copyright © 2020 Anderson and Arora. This is an open-access publication distributed under the terms of the Creative Commons Attribution license.

internal medicine, and neurology), discovered that only 10% could correctly identify all DOAC indications, while 48% felt uncomfortable or very uncomfortable prescribing DOACs.[9] The same group also published a national survey that broadened the scope of health care providers (only 5% were residents or fellows) and included advanced practice nurses, registered nurses, and pharmacists.[10] Of surveyed health care providers, 27% identified all four DOAC indications, while 41% felt uncomfortable or very uncomfortable prescribing DOACs. For both studies, discomfort was mostly attributed to lack of knowledge around DOAC indications, general dosing, renal dosing adjustment, and administration. While it may be tempting to master and preferentially use only one DOAC, with five options now available and the multitude of drug-specific contraindications, cautions, renal dose adjustments, and drug-interactions, providers must familiarize themselves with all DOACs.

We developed a DOAC workshop for the University of Chicago Internal Medicine Residency Program to improve the knowledge, safe prescribing, and confidence around DOACs. Multiple studies have demonstrated anticoagulation knowledge gaps in resident physicians.[11,12] One curriculum coauthor Irsk Anderson serves as the medical director for the Anticoagulant Management Service (AMS), an outpatient-based clinic that longitudinally follows and monitors patients on oral anticoagulation. Irsk Anderson, while serving as the AMS medical director as well as precepting the internal medicine residents in continuity clinic and on the inpatient wards, noted significant and concerning knowledge gaps within the residency program. Residents were frequently requesting curbside consultations for DOAC dosing in chronic kidney disease, use in obese patients, insurance coverage/formulary issues, periprocedural management, and DOAC indications/contraindications. The DOAC workshop was borne out of these knowledge gaps.

As already noted, DOAC knowledge, comfort, and safe prescribing is often lacking across multiple health care providers, including resident physicians. In 2013, Dixon et al published an interprofessional team-based learning module on the topics of general anticoagulation and motivational interviewing.[13] The workshop consisted of mostly internal medicine residents paired with pharmacy residents and psychology graduate students. Three out of 10 readiness assurance test questions were DOAC-specific, but only dabigatran was covered. The authors did not report pre- or postworkshop DOAC knowledge or self-perceived confidence. To our knowledge, no medical resident DOAC-specific curriculum-based interventions have been assessed or published. It seems prudent to target resident physicians in training to ensure adequate DOAC knowledge, comfort prescribing, and management of these high-risk medications upon transition to independent practice.

Methods

Logistics

Forty-nine internal medicine residents (2% PGY 1, 63% PGY 2, and 35% PGY 3) participated in one of four 1-hour workshops between October 2018 and November 2019 (October and December of 2018; January and November of 2019). The content and format of each of the four workshops was the same and the number of residents per workshop ranged from five to 18. The workshop was conducted during their 2-week outpatient block. The workshop could feasibly be used as is or with minor adjustments for medical students rotating through clinical clerkships or residents in other specialties (e.g., surgery, obstetrics/gynecology, neurology, emergency medicine, family medicine). The workshop was conducted in a dedicated resident conference room complete with computer connectivity to a projector and three large-screen display monitors. The workshop was mandatory unless the chief resident approved the absence. No specific prerequisite knowledge or experience was required of the residents, although it was assumed the participants had at minimum heard of DOACs. If the workshop were to be delivered to medical students, it would be prudent to focus on students in their clinical training, assuming they have had education in disease pathophysiology and pharmacology. The workshop facilitator should possess a basic knowledge of the DOACs and their clinic indications. The facilitator slide deck provided the additional knowledge and references for the facilitator(s) to conduct the workshop with their target learners.

Workshop Timeline

5 minutes: Prior to starting the workshop, four preworkshop case-based multiple-choice questions (MCQs) were administered to the internal medicine resident participants (Appendix A). The MCQs were original, created by curriculum co-author Irsk Anderson and linked to the workshop objectives of DOAC indications, dosing, reversibility, and peri-procedural management. The facilitator collected the completed preworkshop MCQs prior to beginning the workshop. Correct answers were not revealed to the workshop participants directly but were covered in the slide deck materials.

5 minutes: The facilitator stated the workshop objectives and reviewed the coagulation cascade pathway, specifically pointing out where in the pathway the direct-thrombin inhibitors and factor Xa inhibitors disrupt coagulation (slide show, Appendix B).

Copyright © 2020 Anderson and Arora. This is an open-access publication distributed under the terms of the Creative Commons Attribution license.

10 minutes: The large group was divided into four smaller workgroups with each workgroup responsible for one unique anticoagulation-specific patient-based case (Appendices C, D, E, F). Each case contained a short patient summary followed by four learning and discussion questions. The residents discussed the case and answered the four questions within their small group. One participant per group was responsible for taking notes for the report-out session. The cases went as follows:

- Case 1 centered around stroke-risk assessment in patients with atrial fibrillation, the benefits and risk of DOACS, DOAC cautions and contraindications, and DOAC dose adjustments.
- Case 2 looked at the determination of individual bleeding risk, DOAC level testing, and DOAC reversal strategies.
- Case 3 focused on DOAC use in elderly patients.
- Case 4 emphasized periprocedural DOAC management.

40 minutes: Each group was given 10 minutes to present their patient case and answers to the learning questions. The report-out exercise was meant to be rich with open discussion led by the facilitator. A slideshow presentation was used to support and enhance the case-relevant material.

5 minutes: Final thoughts and a survey. The postworkshop survey (Appendix G: student survey; Appendix H: facilitator survey with answers) was administered at the end of the workshop and asked the following:

1. Residency year (PGY 1, PGY 2, PGY 3).
2. Rotation through the anticoagulation management clinic (yes or no).
3. Four case-based multiple-choice knowledge questions.
4. Pre- and postworkshop preparedness around five DOAC themes.
5. Overall satisfaction with the workshop.
6. Interest in additional DOAC educational initiatives.

Evaluation

The authors assessed pre- and postworkshop knowledge and self-perceived confidence around DOACs. Four case-based multiple-choice knowledge questions were administered to the resident participants just prior to starting the workshop. Upon completion of the workshop, the residents completed four postworkshop case-based knowledge assessment MCQs. The pre- and postworkshop cases and questions were not identical but touched on three themes: (1) DOAC-specific dosing for atrial fibrillation, (2) DOAC antidotes, and (3) DOAC peri-procedural management. The pre- and postworkshop confidence survey was scored using a 5-point Likert scale (1 = *not confident at all*, 5 = *highly confident*) and assessed confidence in five domains: (1) pros and cons of various DOACs, (2) appropriate initial DOAC dosing, (3) converting to/from DOACs, (4) DOAC contraindications, and (5) DOAC cost and insurance coverage.

Results

Curriculum co-author Irsk Anderson facilitated all four workshops. Survey completion rate was 100%. Five out of 49 residents (10%) had rotated through the AMS prior to attending the workshop. Pre- and postworkshop knowledge-based MCQ data was collected for 33 residents while postworkshop only (no preworkshop) knowledge-based MCQ data was collected for 16 additional residents (pilot group). Thirty-two residents completed the pre- and postworkshop confidence survey while 17 additional residents completed the postworkshop only (no preworkshop) confidence survey (pilot group). The workshop MCQs and pre- and postworkshop confidence surveys were created and structured to assess the resident reaction and learning components of the Kirkpatrick Model of learning evaluation.[14]

Postworkshop DOAC knowledge on the MCQs improved significantly when compared to the preworkshop assessment (see Figure 1; all $p < .003$). The absolute percent of residents answering questions correctly improved by 31%, 31%, 62%, and 81% (questions 1, 2, 3, and 4 respectively) upon completing the workshop. Fifty-three percent of the residents answered all four multiple-choice knowledge questions correctly after the workshop compared to 0% before the workshop ($p < .001$). Postworkshop DOAC confidence scores improved significantly for all five themes (Figure 2; 5-point Likert scale; all $p < .001$ with no overlapping of any 95% confidence intervals). No significance was noted between AMS clinic exposure and pre/post knowledge or confidence. The overall average DOAC workshop satisfaction was 4.2 when asked if they were satisfied with the workshop education (5-point Likert scale; 1 = *strongly disagree*, 5 = *strongly agree*). Eighty-five percent (40/47; two nonresponders) of residents would be interested in additional DOAC-specific training.

Qualitative feedback included, “Would help to put together a decision tree/algorithm/flowsheet for deciding the agent.” When asked if other thoughts or questions, one resident noted, “Learned more so more questions.”

Discussion

DOACs, when prescribed correctly, are at least as effective as warfarin at preventing and treating thromboembolic events with

Copyright © 2020 Anderson and Arora. This is an open-access publication distributed under the terms of the Creative Commons Attribution license.

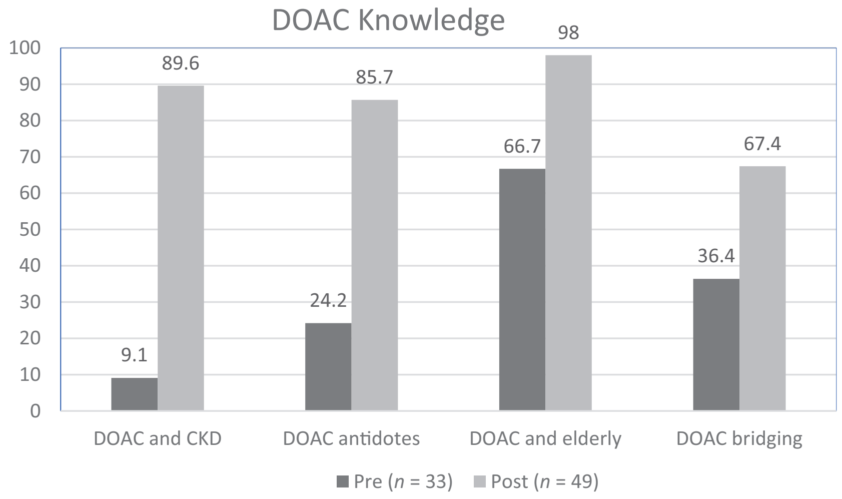

Figure 1. Percentage of residents answering correctly on pretest and posttest multiple-choice questions. For all comparisons, $p < .003$.

an improved overall safety profile. There are nuances, however, related to DOAC prescribing such as dosing by indication, renal-dosing adjustments, DOAC-specific drug-drug interactions, and peri-procedural management guidelines. Resident physicians are responsible for prescribing and monitoring DOACs in both the inpatient and outpatient settings. It is vital, therefore, to improve resident DOAC knowledge not only for their current patients, but for their future patients upon transition to independent practice.

A one-hour workshop significantly improved internal medicine resident DOAC knowledge and self-confidence. Overall workshop satisfaction was high and nearly 9 of 10 residents desired further DOAC training. Covering the material in 1 hour is possible and feasible, however, the depth of the material and subsequent discussions could be improved with either a longer workshop or two to three separate 1-hour workshops. Multiple residents suggested this design at the conclusion of the workshops. A multiple workshop model may be ideal given all postworkshop confidence scores remained less than 4 and the high desire for further DOAC training. The authors are currently working with the resident ambulatory director to spread the workshop across two or three sessions.

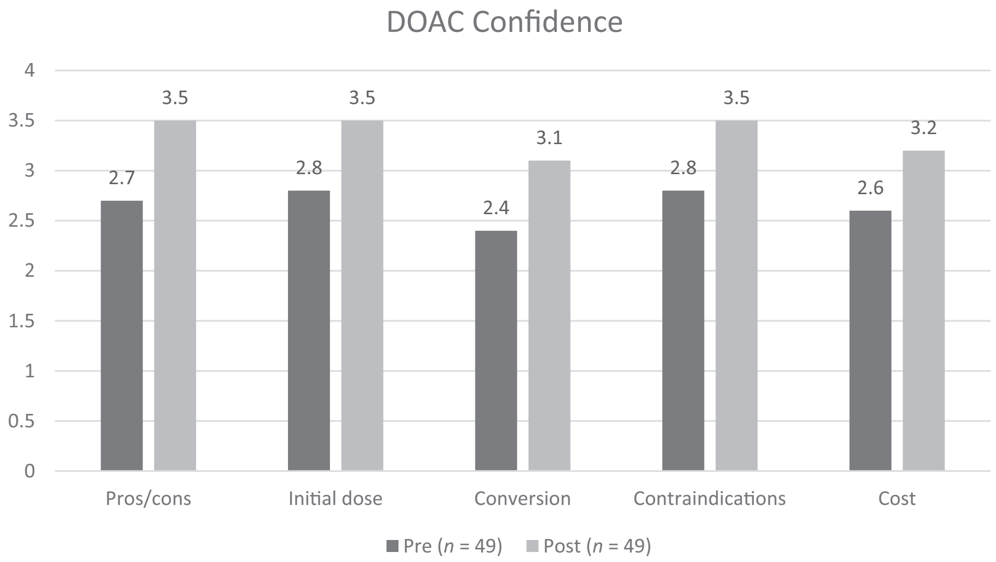

Figure 2. Resident perceived confidence before and after workshop rated on a 5-point Likert scale (1 = *not confident at all*, 5 = *highly confident*). For all comparisons, $p < .001$.

Copyright © 2020 Anderson and Arora. This is an open-access publication distributed under the terms of the Creative Commons Attribution license.

Additional challenges were noted. The small groups occasionally had difficulty staying on track. We would recommend assigning one participant to lead the discussion, one to take notes, and one to report out to the larger group. Adding more faculty moderators could help keep the small groups focused. Some residents realized after the workshop how little they knew before the workshop, a phenomenon referred to as the Dunning-Kruger effect.[14] These students verbalized feeling more confused after the workshop. Possible solutions include longer or multiple workshops to reinforce the material or creating a shared electronic platform for participants to review materials and interact with faculty while promoting longitudinal learning. Attendance was mandatory, however, numerous residents had excusable absences. At least one workshop had very low attendance (<10) making the standard timeline and format challenging. The large-group report-out sessions did not always flow smoothly. We suggest having the small group answer case question A followed by the facilitator reviewing the slides on that topic with open discussion. Next, the small group answers case question B followed by the facilitator reviewing question B slide material and so on.

DOAC research, guidelines, and FDA approvals are constantly changing. A rotating yearly workshop incorporating updated content would allow medicine residents exposure to a higher-level longitudinal experience over their 3-year residency. In addition, hosting the workshop content within an internal private application (e.g., our internal medicine residency uses Slack, a proprietary instant messaging platform that can be used to communicate and share files electronically) would allow facilitators to continuously add/update content that is rapidly accessible as well as facilitate group discussion. DOAC dilemmas come up frequently during inpatient rounds requiring point-of-care decision-making. Having such a resource would fulfill the ACGME Milestone Problem-Based Learning and Improvement item 4: Learning and improvement at the point-of-care.[15]

There are multiple limitations to our workshop. This was a one-institution project with 98% of the internal medicine residents at the PGY 2 or PGY 3 level. It is unclear if more participants were PGY 1 if pre/postworkshop knowledge and confidence differences might be more/less significant. The workshop was not conducted with noninternal medicine resident specialties, medical students, or other allied health professions trainees (e.g., pharmacy, nursing, advanced practice nurses, physician assistants). Such groups may also benefit from a DOAC-specific workshop. The pre- and postworkshop MCQs were not identical, although they did cover the same themes. We did not assess resident DOAC prescribing behaviors or patient outcomes, the more advanced levels of Kirkpatrick's learning evaluation model.[16] Our pre/post confidence survey was administered at the end of the workshop, a practice referred to as the retrospective pre-post method.[17] The small sample sizes allowed us to pair the pre- and post answers by trainee without requiring identifiers. In addition, and based on prior experience, survey completion rates can be low and more time-consuming when administered before and after the workshop. The limitation with this survey design is retrospective recall bias, but studies have shown this to be an effective model that correlates well to the traditional pre/post survey and may reduce response shift bias.[17]

The authors plan to expand the workshop to additional resident specialties, medical students, and allied health trainees and practitioners. We are currently designing a study to further assess internal medicine resident DOAC prescribing behaviors and patient outcomes in the inpatient and outpatient practice environments.

Appendices

A. Preworkshop MCQ Students.docx
B. DOAC PowerPoint.ppt
C. DOAC Indications and Dosing Case.docx
D. DOAC Monitoring and Reversal Case.docx
E. DOAC Dosing Elderly Case.docx
F. DOAC Peri-procedural Case.docx
G. Postworkshop MCQ and Confidence Survey Students.docx
H. Postworkshop MCQ Facilitators.docx

All appendices are peer reviewed as integral parts of the Original Publication.

Irsk Anderson, MD: Assistant Professor, Department of Medicine, University of Chicago Pritzker School of Medicine

Vineet M. Arora, MD: Professor, Department of Medicine, University of Chicago Pritzker School of Medicine

Acknowledgments
We would like to acknowledge Drs. Amber Pincavage and Shannon Martin for their assistance and support.

Disclosures
None to report.

Funding/Support
None to report.

Copyright © 2020 Anderson and Arora. This is an open-access publication distributed under the terms of the Creative Commons Attribution license.

Ethical Approval
Reported as not applicable.

Disclaimer
The authors' views expressed in the submitted article are their own and not an official position of the institution.

References

1. Huisman MV, Rothman KJ, Paquette M, et al. The changing landscape of stroke prevention in AF: findings from the GLORIA-AF registry phase 2. *J AM Coll Cardiol*. 2017;69(7):777-785. https://doi.org/10.1016/j.jacc.2016.11.061
2. Kearon C, Aki EA, Ornelas J, et al. Antithrombotic therapy for VTE disease. CHEST guideline and expert panel report. *Chest*. 2016; 149(2):315-352. https://doi.org/10.1016/j.chest.2015.11.026
3. Kirchhof P, Benussi S, Kotecha D, et al. 2016 ESC Guidelines for the management of atrial fibrillation developed in collaboration with EACTS. *Eur Heart J*. 2016;37(38):2893-2962. https://doi.org/10.1093/eurheartj/ehw210
4. January CT, Wann LS, Calkins H, et al. 2019 AHA/ACC/HRS focused update of the 2014 AHA/ACC/HRS guideline for the management of patients with atrial fibrillation. *J Am Coll Cardiol*. 2019;74(1):104-132. https://doi.org/10.1016/j.jacc.2019.01.011
5. Vinter N, Linder M, Andersen M, et al. Classification and characteristics of on-label and off-label apixaban use in Denmark and Sweden. *Pharmacoepidemiol Drug Saf*. 2019;28(6):867-878. https://doi.org/10.1002/pds.4778
6. Schwartz JB, Merrill S, de Leon N, Thompson A, Fang MC. Dosing accuracy of direct oral anticoagulants in an academic medical center. *J Hosp Med*. 2017;12(7):544-550. https://doi.org/10.12788/jhm.2769
7. Steinberg BA, Shrader P, Thomas L, et al. Off-label dosing of non-vitamin K antagonist oral anticoagulants and adverse outcomes: the ORBIT-AF II registry. *J Am Coll Cardiol*. 2016; 68(24):2597-2604. https://doi.org/10.1016/j.jacc.2016.09.966
8. Arbel R, Hammerman A, Sergeinko R, et al. Abstract 36: direct oral anticoagulant underdosing increases rates of stroke and myocardial infarction with no reduction in bleeding rate. *Stroke*. 2019;50:A36. https://doi.org/10.1161/str.50.suppl_1.36
9. Piran S, Schulman S, Salib M, Delaney J, Panju M, Pai M. Direct oral anticoagulants in the real world: insights into Canadian health care providers' understanding of medication dosing and use. *Can J Gen Intern Med*. 2017;12(3):23-27. https://doi.org/10.22374/cjgim.v12i3.201
10. Piran S, Schulman S, Panju M, Pai M. Oral anticoagulant dosing, administration, and storage: a cross-sectional survey of Canadian health care providers. *J Thromb Thrombolysis*. 2018;45(1)180-185. https://doi.org/10.1007/s11239-017-1585-y
11. Yazdan-Ashoori P, Oqab Z, McIntyre WF, et al. How do family medicine residents choose an anticoagulation regimen for patients with nonvalvular atrial fibrillation? *Prim Health Care Res Dev*. 2017;18(5):472-481. https://doi.org/10.1017/S1463423617000196
12. Moulson N, McIntyre WF, Oqab Z, et al. The anticoagulation choices of internal medicine residents for stroke prevention in non-valvular atrial fibrillation. *Postgrad Med J*. 2017;93(1100): 308-312. https://doi.org/10.1136/postgradmedj-2016-134159
13. Dixon DL, Lyles A, Sicat B, Lee B, Rybarczyk B, Lord B. Interprofessional team-based learning module: anticoagulation. *MedEdPORTAL*. 2013;9:9334. https://doi.org/10.15766/mep_2374-8265.9334
14. Kruger J, Dunning D. Unskilled and unaware of it: how difficulties in recognizing one's own incompetence lead to inflated self-assessments. *J Pers Soc Psychol*. 1999;77(6):1121-1134. https://doi.org/10.1037//0022-3514.77.6.1121
15. The Internal Medicine Milestone Project. The Accreditation Council for Graduate Medical Education and the American Board of Internal Medicine; 2015. Accessed August 22, 2020. https://www.acgme.org/Portals/0/PDFs/Milestones/InternalMedicineMilestones.pdf
16. Kirkpatrick DL. *Evaluating Training Programs: The Four Levels*. Berrett-Koehler; 1994.
17. Bhanji F, Gottesman R, de Grave W, Steinert Y, Winer LR. The retrospective pre-post: a practical method to evaluate learning from an educational program. *Acad Emerg Med*. 2012;19(2): 189-194. https://doi.org/10.1111/j.1553-2712.2011.01270.x

Received: December 19, 2019
Accepted: April 16, 2020
Published: September 30, 2020

Copyright © 2020 Anderson and Arora. This is an open-access publication distributed under the terms of the Creative Commons Attribution license.
